# ECE vs DISP Mechanisms in Anodic Electrolysis of Benzyl Alcohols: Computational Prediction of Microscopic Rate Constants

**DOI:** 10.1021/acs.jpcc.5c04347

**Published:** 2025-08-28

**Authors:** John H. Hymel, Jesse G. McDaniel

**Affiliations:** School of Chemistry and Biochemistry, 1372 Georgia Institute of Technology, Atlanta, Georgia 30332-0400, United States

## Abstract

The heterogeneous nature of electrochemical reactions entails unique kinetic control of product yield/selectivity as compared with corresponding homogeneous oxidation/reduction reactions. In direct electrolysis, subsequent elementary steps following the initiating electron transfer may also occur heterogeneously at the electrode surface or homogeneously within the bulk electrolyte, often via a disproportionation step for secondary electron transfer; kinetic control of this branching may have important consequences for product selectivity/yield, due to differences in lifetimes of reactive radical intermediates. In this work, we use computer simulations to predict microscopic rate constants governing the heterogeneous “ECE” electrochemical oxidation of para-methoxybenzyl alcohol to its corresponding aldehyde at a working carbon anode within an aqueous electrolyte. Molecular dynamics simulations are conducted to model the full electrochemical cell at atomistic resolution under conditions approximating controlled potential electrolysis, from which rate constants are predicted via a combination of direct dynamics and free energy sampling methods. Density functional theory-based quantum mechanics/molecular mechanics (DFT-QM/MM) simulations are performed to predict free energy barriers for deprotonation of the cation radical intermediate within the electrical double layer environment. We demonstrate how strong solvophobic forces lead to residence times of ten(s) of nanoseconds for the electrogenerated cation radical intermediates to reside within the anodic double layer, and the relative deprotonation rate is a key factor dictating the heterogeneous vs homogeneous reaction branching. We predict a compelling double-layer modulation for the cation radical deprotonation rate with NaOAc aqueous electrolyte, arising from a combination of preformed “encounter pairs” via ionic interactions and reduction in activation barrier via stereoelectronic effects. Our computational study of this prototypical electrolysis reaction illustrates the substantial role of reaction conditions (solvent, electrolyte, and overpotential) on the microscopic rate constants that kinetically control the reaction pathway/outcome.

## Graphical abstract

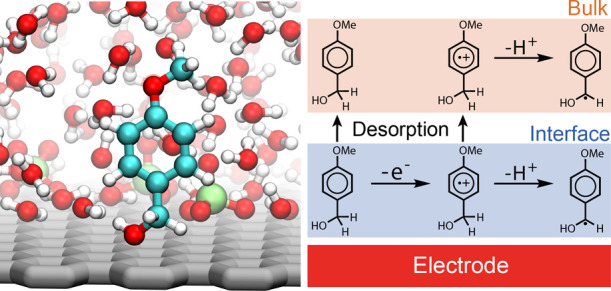

## 1 Introduction

Direct electrolysis represents a straightforward protocol in organic electrosynthesis, in which substrates are converted to reactive radical or ionic intermediates by direct electron transfer at the working electrode, with the electrogenerated intermediates spontaneously reacting to products often in the absence of explicit catalysts. This approach is advantageous due to its simplicity, scalability, and suitability for practical industrial processes, including continuous-flow cell reactors. Electrosynthesis encompasses a wide scope of useful synthetic organic transformations, as described in many excellent review articles;^1–12^ some notable examples include the Kolbe reaction,^7,13–15^ the Baizer process for adiponitrile synthesis,^16–19^ aryl coupling reactions,^4,5,14^ and intramolecular cyclization reactions.^7,8,12^ The primary challenge is that for poorly designed electrolysis conditions (e.g., substrate, solvent, electrolyte, overpotential, etc.), uncontrolled radical side reactions can lead to low selectivity/yield of the target synthetic product. Exploiting electroauxiliary groups,^1^ electrochemical mediators (i.e., indirect electrolysis),^20^ or molecular catalysts^21^ are all important ways to control and/or direct the inherent radical reactions. The focus in this work is on understanding how solvophobic forces at the heterogeneous electrochemical interface can play a critical role in dictating the kinetic competition of various reaction pathways, as important for product selectivity and yield.

Compared with the corresponding chemical (or photochemical) oxidation/reduction reactions, electrolysis reactions are unique in that they are *heterogeneous*, being initiated at the electrode surface and within the electrical double layer (EDL) formed at the working electrode. The heterogeneous nature of the process introduces additional and unique kinetic considerations, in addition to the chemical kinetics of the radical reaction(s) that are present in corresponding homogeneous reactions.^22^ Because electrochemical reactions often proceed via sequential *electrical* “E” and *chemical* “C” elementary steps, an important question is which (or all?) of these steps occur *heterogeneously* at the electrode surface, with the possibility that later steps could proceed *homogeneously* if intermediates diffuse away from the electrode and out of the EDL. For explicit context, we consider the common example of an electrochemical reaction proceeding via an initial *electrical*-*chemical*-*electrical* or “ECE” pathway;^22,23^ the elementary steps for an anodic/oxidative ECE electrolysis reaction for the case with a chemical step corresponding to deprotonation are shown below

$$\mathrm{HA}\overset{k_{\mathrm{ET}}}{⇌}{\mathrm{HA}}^{+•}+e^{-}$$ 1

$${\mathrm{HA}}^{+•}\overset{k_{C}}{⇌}A^{•}+H^{+}$$ 2

$$A^{•}\overset{k_{\mathrm{ET}}}{⇌}A^{+}+e^{-}$$ 3

The substrate label “HA” explicitly indicates the proton that will be lost during the sequence of oxidation steps. The first step represents heterogeneous electron transfer to the electrode, providing the thermodynamic driving force for the entire electrosynthesis reaction via the working potential applied to the electrode. For outer-sphere electron transfer processes, the resulting intermediate “HA^+•^” is a highly reactive radical ion, with remaining steps being thermodynamically downhill and thus subject only to kinetic control. The kinetics of the intermediate chemical step, in this case, proton transfer, is a key consideration for whether the remaining steps proceed heterogeneously or homogeneously. If this chemical step (with rate constant “*k*_*C*_”) is fast relative to the residence time of the substrate/intermediate near the electrode surface,^23^ then this step and the subsequent electron transfer to form intermediate “A^+^” are likely to occur heterogeneously at the electrode surface. Conversely, if the chemical step is slow, the intermediate “HA^+•^” will diffuse into the bulk electrolyte before reacting homogeneously; because the resulting intermediate “A^•^” will then be far from the electrode, the second electron transfer must then occur homogeneously via the disproportionation (DISP) mechanism shown below^23^

$$A^{•}+{\mathrm{HA}}^{+•}\overset{k_{\mathrm{ET}}}{⇌}A^{+}+\mathrm{HA}$$ 4

Note that such homogeneous electron transfer is usually thermodynamically favorable, given that the initial substrate “HA” typically has a higher oxidation potential than that of the intermediate “A^•^”. However, the homogeneous electron transfer in eq 4 is second order in electrogenerated intermediates “HA^+•^” and “A^•^” that may be present at low concentrations, and it may compete with side reactions involving the radical intermediate(s).

The focus of our present work is to investigate the above ECE versus DISP mechanistic pathways for the case study of anodic electrolysis of benzyl alcohol substrates, with computational predictions of the relevant microscopic rate constants. Electrochemical oxidation of benzyl alcohols and related substrates to their corresponding aldehydes via direct electrolysis has a long history,^14,24–27^ with recent reports of extremely high yield/selectivity for these electrolysis reactions conducted within flow cell reactors.^28^ Mechanistic investigation is warranted given the broader general significance of oxidative transformations of aryl substrates via direct electrolysis, and realizing that various pathways may be kinetically competitive depending on reaction conditions. For example, other important transformations of aryls via anodic electrolysis include oxidative substitution of aryl rings and side chains,^29–37^ aryl coupling reactions,^4,5,33,38–44^ and selective alcohol protection or deprotection.^45^ Many of these transformations are thought to proceed via aryl cation radical intermediates, and thus, elucidating the relevant heterogeneous reaction kinetics of these intermediates is of general importance.

The investigation of heterogeneous reaction kinetics for anodic benzyl alcohol transformations has both practical synthetic importance for benzyl aldehyde production and serves as a prototype for the mechanistic understanding of broader classes of electrosynthesis reactions that proceed via similar ECE mechanisms (eq 1). In terms of practical importance, empirical evidence suggests that the target aldehyde yield from electrolysis of benzyl alcohols depends strongly on reaction conditions. For instance, continuous-flow electrochemical reactors employing carbon-based anodes have shown significantly enhanced yields compared to batch electrolysis in an undivided cell.^28^ In addition, reported yields from this recent study were substantially higher than earlier studies that utilized different electrolysis conditions.^14,24–27^ These empirical findings suggest that kinetic competition from side reactions can lead to lower selectivity and yield for unoptimized conditions. In this regard, whether the benzyl alcohol oxidation follows the heterogeneous ECE or homogeneous DISP pathway (eqs 1 and 4) may have important implications for yield/selectivity, as related to the lifetime of the reactive benzyl radical intermediate and thus its propensity for side reactions.^46^ The benzyl radical intermediate, formed after loss of one electron and one proton, could potentially undergo numerous side reactions, including hydrogen atom abstraction or dimerization,^22^ and/or coupling with benzyl aldehyde product to a 1,2-diol.^47–50^ If the entire electrosynthesis reaction occurs heterogeneously via the ECE pathway (eq 1), such side reactions may be minimized, given presumably rapid oxidation of the benzyl radical at the anode resulting from a high overpotential (vide infra). In contrast, the DISP pathway may lead to longer lifetimes of the benzyl radical intermediate (and thus be more prone to side reactions), given that the second oxidation step occurs via second-order kinetics (eq 4) depending on concentrations of electrogenerated intermediates.

Similarly, benzylic alcohol oxidation serves as a prototype reaction to investigate anodic electrolysis mechanisms for which cation radical deprotonation acts as a key intermediate (and possibly a rate-limiting) step. While original work on ECE vs DISP mechanisms by Savéant and co-workers focused primarily on cathodic electrolysis reactions,^46,51–53^ Pons and co-workers have investigated ECE vs DISP mechanisms for anodic electrolysis of methylbenzenes.^54,55^ This latter process proceeds through a cation radical deprotonation step, followed by a second oxidation step and solvent trapping of the carbocation; the initial ECE mechanistic steps are thus analogous to the benzylic alcohol oxidation considered here. More generally, the deprotonation of alkylaromatic cation radicals has been investigated within numerous contexts, given the unique and practical aspects of these organic acids.^56–65^ Of note is the fact that despite being very strong acids with typical p*K*_a_’s ∼ −5 to −25,^56,57^ deprotonation of alkylaromatic cation radicals is often governed by moderate activation barriers of ∼0.3–0.6 eV for deprotonation to either base or to solvent.^60,61,63^ In some cases, deprotonation rates correlated with the p*K*_a_ of the cation radical,^63^ but not in other cases,^60^ and deprotonation rates were found to correlate with homolytic bond dissociation energies.^60,61^ The mechanism of alkylaromatic cation radical deprotonation has been described as a “concerted electron–hydrogen atom transfer,” ^60,61^ while other work has demonstrated a substantial stereoelectronic contribution to the deprotonation barriers of these species.^58,59,62,63^ In this study, we will present computational predictions that suggest that the stereoelectronic contribution to the deprotonation barrier of benzylic cation radicals is substantial and can be modulated by the heterogeneous nature of the electrode interface.

In this work, we computationally investigate the heterogeneous reaction kinetics for the electrochemical oxidation of para-methoxybenzyl alcohol (PMBA) to its corresponding aldehyde at a carbon working electrode within an aqueous electrolyte. We predict/characterize several key microscopic rate constants that dictate whether the electrolysis reaction proceeds via ECE vs DISP pathways, focusing on the residence time of electrogenerated intermediates at the working electrode surface and the deprotonation rate of the cation radical intermediate. The other key microscopic step, namely, the second heterogeneous electron transfer, is assumed to be rapid given the substantial overpotential at the working electrode for the oxidation of the (neutral) benzyl radical intermediate (as predicted computationally). Our computational characterization/predictions incorporate an atomistic description of the full electrochemical cell with electrode held at working potential/charge, employing both classical molecular dynamics (MD) and density functional theory (DFT)-based quantum mechanics/molecular mechanics (QM/MM) free energy simulations, with the latter utilized to compute deprotonation reaction free energy profiles.

We illustrate how the electrical double layer (EDL) modulates the microscopic rate constants governing the ECE pathway through both solvophobic and electrostatic interactions. Solvophobic forces within the aqueous electrolyte result in substantial free energy minima of ∼30 kJ/mol for the benzyl alcohol to associate/adsorb to the carbon electrode at low surface charge. This attractive substrate/electrode association remains but is substantially modulated, as the electrode is charged to a higher working potential and the substrate is oxidized to the cation radical intermediate. The residence time of the electrogenerated cation radical at the working electrode is predicted to be on the order of ten ns, but is highly variable with the precise electrode charge/overpotential. This implies that if the rate constant *k*_*C*_ for deprotonation of the benzyl cation radical intermediate is *k*_*C*_ ≥ 10^9^ s^–1^ (i.e., pseudo first order), the heterogeneous ECE pathway is likely, whereas the DISP pathway is dominant for slower deprotonation kinetics of *k*_*C*_ ≤ 10^7^ s^–1^. DFT-QM/MM free energy simulations are utilized to compute reaction barriers for the benzyl cation radical deprotonation within aqueous LiClO_4_ and NaOAc electrolytes, for both homogeneous and heterogeneous reactions, which provide transition state theory (TST) estimates of the deprotonation rate constant, *k*_*C*_. These computed rate constants provide a detailed microscopic perspective of how the underlying reaction conditions (e.g., solvent, electrolyte, electrode type, working potential, etc.) kinetically mediate the ECE vs DISP pathway for the electrochemical reaction. We discuss a compelling prediction of a substantially reduced free energy barrier for the deprotonation reaction, as occurring heterogeneously within the EDL of the NaOAc electrolyte. This is based on the unique EDL environment near the electrode, in which specific interactions with the acetate ion(s) alter the stereoelectronic contribution to the deprotonation barrier, reducing the barrier by ∼20–25 kJ/mol.

Our manuscript is organized as follows: In Section 2, we discuss the computational methods and system construction for both classical and QM/MM MD simulations of the benzylic alcohol substrate (PMBA) within the electrochemical cell under working conditions. The free energy sampling techniques are discussed, including the (difficult) deprotonation free energy simulations, which must adequately sample the (localized) transition state despite the tendency of the product hydronium ion to delocalize via Grotthuss transport. Results are presented in Section 3, first discussing the anodic double layer structure and substrate-electrode association free energies in Section 3.1. In Section 3.2, we present computational predictions for the desorption kinetics of the PMBA substrate and its electrogenerated cation radical intermediate from the anode surface at different electrode surface charge/working potential. We then present and discuss results for DFT-QM/MM computed free energy profiles for deprotonation of the cation radical intermediate for both homogeneous and heterogeneous pathways; Section 3.3 presents results for the aqueous LiClO_4_ electrolyte, and Section 3.4 presents results for the NaOAc electrolyte in which a unique and compelling double layer modulation of the reaction free energy barrier is observed.

## 2 Methods

### 2.1 Electrochemical Cell Simulation Setup

Classical molecular dynamics (MD) simulations were performed to model the electrochemical cell under the working conditions. The electrochemical cell consisted of two carbon electrodes, each modeled as rigid graphene sheets with lattice vectors of length 4.93 nm and at a 120° angle, separated by the ∼12 nm region of aqueous electrolyte. The system was modeled with 3D periodic boundary conditions (PBC), and a large vacuum gap of 25 nm was included along the *z*-axis to prevent interactions between periodic replicas of the cell along this dimension. The aqueous electrolyte consisted of 8000 water molecules, with an approximately 0.4 M concentration of ions. Both lithium perchlorate (LiClO_4_) and sodium acetate (NaOAc) electrolytes were studied, with respective systems containing either 75 LiClO_4_ or 80 NaOAc ion pairs. Sections 3.1–3.3 discusses results based on the aqueous LiClO_4_ electrolyte, and Section 3.4 discusses results from the aqueous NaOAc electrolyte system. Because the bulk electrolyte region is finite, charging the electrodes/double layers leads to depletion of ions from the bulk and non-negligible changes in bulk ion concentration. For the various electrode surface charge densities studied, we computed the final ion concentrations in the bulk region of the electrochemical cell after equilibration; these are given in Figure S1 of the Supporting Information and range from 0.2–0.4 M. For brevity, we will refer to the “0.4 M” electrolyte systems throughout the manuscript, but it is understood that precise concentrations correspond to those in Figure S1.

The initial electrochemical cell was constructed by generating electrolyte atom positions using the PackMol^66^ software with the electrodes at the boundary region. The system is purposely generated at low density to avoid unphysically large repulsive energies that would lead to simulation crashes. The system density is then equilibrated with a hybrid molecular dynamics/Monte Carlo (MD/MC) protocol, in which MD integration of electrolyte atom positions is interspersed between MC moves of (one of) the rigid graphene electrodes along the *z*-axis of the electrochemical cell.^67^ This hybrid MD/MC equilibration simulation is run for 10 ns, and it is verified that the electrolyte density between the electrodes is equilibrated. Simulations are then conducted at various electrode surface charge densities, and in each case, the simulation is equilibrated for 50 ns in the NVT ensemble when a new surface charge density is applied to the electrodes. Each electrode is modeled at constant charge, distributed uniformly across all carbon atoms of the graphene electrode surface; additional details are given in the Supporting Information. We note that neglect of explicit image charges in the modeling may introduce some error in our predictions; however, it is expected that image charge interactions may be largely screened by the solvent.^68^ All simulations were conducted at 300 K using a Langevin thermostat with a friction coefficient of 1 ps^–1^ and a 1 fs time step. The particle mesh Ewald (PME) approach^69^ was utilized for long-range electrostatics, and van der Waals interactions were truncated at a 1.4 nm cutoff distance. All classical MD simulations were conducted utilizing the OpenMM v7.7 software package.^70^

The force field utilized within the classical MD simulations is as follows. The SPC/E model was used for water,^71^ with OPLS-AA “compatible” parametrizations for the other system components.^72^ Specifically, force field parameters for the electrolyte ions Li^+^, ClO_4_^–^, Na^+^, and acetate were taken from prior studies.^73–75^ Graphene carbon parameters were taken from the work of Schyman and Jorgensen.^76^ Force field parameters for the PMBA substrate were generated using the Ligpargen utility.^77^ To model the PMBA cation radical, the Lennard-Jones and bonded parameters were taken to be the same as the neutral substrate, while atomic partial charges were fit for the specific oxidation state. Atomic charges were fit based on geometry optimizations and single-point calculations at the PBE0/6–31G* level of theory, conducted using the Psi4 software.^78^ Distributed multipole analysis (DMA) was conducted,^79,80^ followed by subsequent electrostatic potential fitting of each of the DMA sites to generate atomic charges, based on the method described by Ferenczy and co-workers.^81,82^ The parametrized atomic charges for the different oxidation states of PMBA are given in Table S1 of the Supporting Information. Example simulation input files, including force field parameters needed to run the MD simulations, are included in the Supporting Information.

### 2.2 Electrode/Substrate PMFs and Desorption Rate Constant Computations

To investigate electrode/substrate association, potentials of mean force (PMFs) were computed for the separation distance “*r*_anode/PMBA_” between the PMBA substrate and working electrode (anode) at various electrode surface charges. PMFs were generated by umbrella sampling followed by analysis using the weighted histogram analysis method (WHAM).^83^ Umbrella sampling was conducted using the PLUMED2 software,^84^ with umbrella potentials applied to the center of mass (COM) of the PMBA substrate (or corresponding cation radical intermediate). For each PMF, 19 umbrella sampling windows were placed at 0.5 Å intervals spanning a distance range of 3.0 ≤ *r*_anode/PMBA_ ≤ 12.0 Å, with a harmonic force constant of 20 kJ/mol/Å^2^ for each umbrella. Simulations were initially “steered” to the desired *r*_anode/PMBA_ separation distance over 100 ps, followed by 20 ns of production sampling per window, resulting in a total of 380 ns of production simulation to generate each PMF. For the aqueous LiClO_4_ electrolyte, PMFs were computed for both the neutral/oxidized PMBA substrate for 16 different electrode surface charge densities, ranging from 0 to 30 μC/cm^2^, in 2 μC/cm^2^ increments. For the aqueous NaOAc electrolyte, this single PMF was computed for the oxidized PMBA substrate at a working electrode charge of 14 μC/cm^2^. The motivation for these choices is discussed in Section 3.1.

Residence times for the neutral and oxidized PMBA substrates near the working electrode surface were computed from direct (unbiased) MD simulations. First-order rate constants for desorption from the electrode surface are then given as the inverse of the computed residence time. The desorption process was defined based on the PMF free energy profiles for the substrate-electrode distance, *r*_anode/PMBA_. The adsorbed state was taken to be *r*_anode/PMBA_ = 3.5 ± 0.25 Å for neutral PMBA and *r*_anode/PMBA_ = 5.0 ± 0.25 Å for oxidized PMBA, corresponding to the observed PMF minima. Desorption was defined to occur when the center of mass of PMBA exceeded 15 Å from the electrode surface, which is the region where the PMF plateaus to a constant value (vide infra). To improve sampling, a half-harmonic upper wall with a force constant of 500 kJ/mol/Å^2^ was placed at 20 Å from the electrode to prevent substrate diffusion into the bulk during these simulations. Simulations were performed within LiClO_4_ electrolyte at five different anode surface charge densities of 0, 6, 14, 20, and 28 μC/cm^2^. At each electrode charge, ten independent 250 ns (unbiased) MD simulations were run. For the σ = 0 μC/cm^2^ electrode charge, where desorption events were rare, all ten replicas were extended for an additional 250 ns. More details of the procedure can be found in the Supporting Information, and we note that similar approaches have been reported in the literature.^85,86^

### 2.3 QM/MM Molecular Dynamics Simulations

DFT-based QM/MM molecular dynamics simulations were performed to compute free energy profiles for deprotonation of the PMBA radical cation by either water or acetate, both in bulk solution and at the electrochemical interface. All QM/MM simulations were conducted utilizing the PyDFT-QMMM software,^87^ which interfaces Psi4 ^78^ and OpenMM^70^ as QM and MM engines, respectively. All QM/MM simulations were initialized from equilibrated classical MD configurations (Section 2.1) at an electrode surface charge density of 14 μC/cm^2^. The QM region included the PMBA radical cation and the base (either H_2_O or OAc^–^) as well as various numbers of solvent water molecules (specifics will be given in the context of results discussion). The QM region was treated at the B3LYP-D3/def2-SVP level of theory,^88,89^ while the remaining solvent/electrolyte and electrode atoms were modeled classically according to the force field described in Section 2.1. The only force field difference is that the flexible SPC/Fw water model^90^ is utilized in the QM/MM simulations. The QM/MM interaction was modeled with electrostatic embedding, employing a truncation cutoff of 14 Å (on a molecule-by-molecule basis) for incorporating MM partial charges into the Kohn–Sham Hamiltonian. QM/MM MD simulations were performed in the NVT ensemble, utilizing a Langevin thermostat with a friction of 5 ps^–1^. Example simulation input files needed to run the QM/MM MD simulations are included as Supporting Information.

Umbrella sampling was utilized in combination with QM/MM MD simulations to generate free energy profiles for cation radical deprotonation reactions. The reaction coordinate was a proton transfer coordinate, defined as the difference in bond distances between the acidic proton and the donor and acceptor atoms, specifically

$$R_{\mathrm{PT}}=d_{C-H^{+}}-\min(d_{H^{+}-O})$$

with *d*_C–H^+^_ the acidic hydrogen bond distance on the −CH_2_ group of the PMBA cation radical, and *d*_H^+^–O_ the distance between acidic hydrogen and oxygen atom of acceptor (either H_2_O or OAc^–^). The applied umbrella potentials utilized a force constant of 750 kJ/mol/Å^2^, with starting configurations initialized using 2 ps of steered MD, followed by 16–20 ps of production sampling per window. Since both hydrogen atoms of the −CH_2_ group are acidic, we chose one for the deprotonation reaction and applied a half-harmonic wall restraining the other hydrogen (at 1.2 Å) to prevent it from deprotonating.

When the PMBA cation radical is deprotonated in aqueous media, the acidic proton (hydronium ion) is rapidly delocalized in the solvent via Grotthuss diffusion. This requires special treatment within QM/MM free energy sampling. First, since only “QM” water molecules can participate in the Grotthuss bond formation/breaking process, the QM region is chosen to encompass a “shell” of 6–8 water molecules surrounding the acidic proton. The “Flexible Inner Ensemble Separator” (FIRES) restraint/method^91^ is then used to prevent diffusion/switching between MM and QM water molecules within this solvation shell around the acidic proton. An additional restraint is imposed to localize the acidic proton within the QM region consisting of the substrate and the QM water solvation shell. We employed an auxiliary collective variable (CV) based on the Voronoi polyhedra method developed by Parrinello and co-workers.^92,93^ This CV measures the distance between the benzylic −CH_2_ carbon of PMBA and the acidic proton, which is identified through a continuous, differentiable topological criterion. The Voronoi-based CV facilitates tracking of the location of the acidic proton independent of molecular topology. We then apply a half-harmonic upper wall at 3.5 Å along this CV to prevent the acidic proton from diffusing out of the QM solvation shell. The application of this restraint was limited to simulations involving water as the proton acceptor. For deprotonation by acetate, the proton remains localized on a single acceptor atom, and no such restraint was necessary. All of these biased sampling procedures utilized the PLUMED2 software,^84^ with free energy profiles constructed utilizing WHAM.^83^ Additional details of this procedure are given in the Supporting Information.

The final additional detail is that Lennard-Jones parameters for the benzylic −CH_2_ group were tuned specifically for these deprotonation simulations. This was to ensure that when the acidic −CH_2_ proton transferred to water, the force field would appropriately describe the hydronium ion solvation (without switching the force field parameters based on molecular topology). We set the benzylic −CH_2_ hydrogen parameters to those of the SPC/E water model,^94^ and tuned the carbon LJ parameters to match the intermolecular interactions as described by the original OPLS-AA parametrization of the −CH_2_ group. Specifically, the new carbon LJ parameters were scaled to reproduce the radial distribution function between the −CH_2_ group and the water solvent from the original OPLS-AA parameter set. Details and benchmarks of this parameter tuning are given in the Supporting Information.

Pseudo-first-order rate constants for deprotonation were estimated using transition state theory via the Eyring equation:

$$k_{C}=\frac{k_{B}T}{h}\exp\left(-\frac{\Delta G^{‡}}{k_{B}T}\right)$$ 5

where *k*_*C*_ is the rate constant for deprotonation, *k*_*B*_ is Boltzmann’s constant, *h* is Planck’s constant, and *T* is the temperature. The free energy barrier Δ*G* ^‡^ is computed from the QM/MM free energy simulations, as described above.

## 3 Results

We investigate the electrochemical oxidation of para-methoxybenzyl alcohol (PMBA) to the corresponding aldehyde, a reaction that proceeds via a net two-electron and two-proton process, as shown schematically in Figure 1. We assume that the electron and proton transfer steps proceed sequentially, and not through a concerted proton-coupled electron transfer process.^95–97^ This is because when the anode is held at working potential (or positive overpotential), the initial (heterogeneous) outer-sphere electron transfer is thermodynamically neutral/favorable in free energy, and kinetic barriers associated with solvent reorganization energy are dramatically reduced near the electrode surface.^98,99^ The full electrochemical process will involve an additional cathodic half reaction (which is typically hydrogen evolution), which we do not consider here, as the focus is purely on the anodic electrosynthesis reaction. We note that experimental studies utilizing electron paramagnetic resonance (EPR) have confirmed the existence of the neutral radical intermediate within the proposed mechanism for the anodic transformation of benzylic alcohols to their corresponding aldehydes.^28^

**Figure 1:**
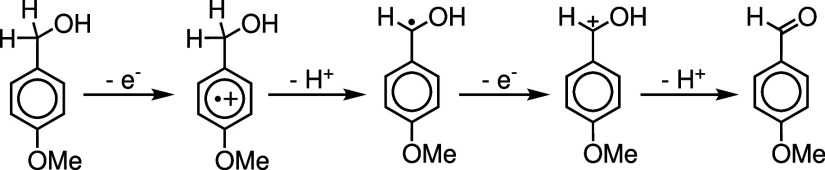
Schematic depicting the mechanistic steps and intermediates involved in the electrochemical oxidation of para-methoxybenzyl alcohol (PMBA) to the corresponding aldehyde.

The proposed mechanism in Figure 1 is an “ECEC” electrochemical process with alternating electron transfer (oxidation) and deprotonation steps. Quantum chemical calculations predict (vide infra) that the last deprotonation step is rapid, and we thus primarily focus on the initial “ECE” steps of the mechanism (and refer to the process as “ECE” henceforth). The initiating electron transfer must occur heterogeneously at the anode surface, but the following steps (deprotonation of the cation radical and second electron transfer/oxidation) could occur either heterogeneously or homogeneously, depending on the relative kinetics. Figure 2 depicts this possible branching between the heterogeneous “ECE” pathway, compared to the homogeneous “DISP” pathway, the nomenclature for the latter stemming from the disproportionation step for the second electron transfer. The PMBA substrate is denoted with the general notation “HA”, with labeling of the reaction intermediates consistent with eqs 1–4. This diagram emphasizes the key kinetic steps governing these competing mechanisms, including rate constants for electron transfer (*k*_ET_) and deprotonation (*k*_*C*_), either of which could occur heterogeneously or homogeneously (“Het” or “Hom” superscripts, respectively). The labels “ET,1” and “ET,2” are used to differentiate between heterogeneous electron transfer rate constants for the first and second oxidation. The branching between the “ECE” vs “DISP” pathway depends on kinetic competition between these mechanistic steps and the process of intermediate desorption from the heterogeneous electrode surface. While textbook treatments of the ECE vs DISP branching focus on comparison of mass transport (e.g., diffusion coefficients) to chemical rates,^22,23^ such analysis omits consideration of the large solvophobic forces that give rise to substantial free energies of “adsorption” for organic substrates to carbon electrodes in aqueous electrolytes. These solvophobic forces, adsorption free energies, and kinetics for substrate desorption from the working electrode are discussed in detail in Sections 3.1 and 3.2.

**Figure 2:**
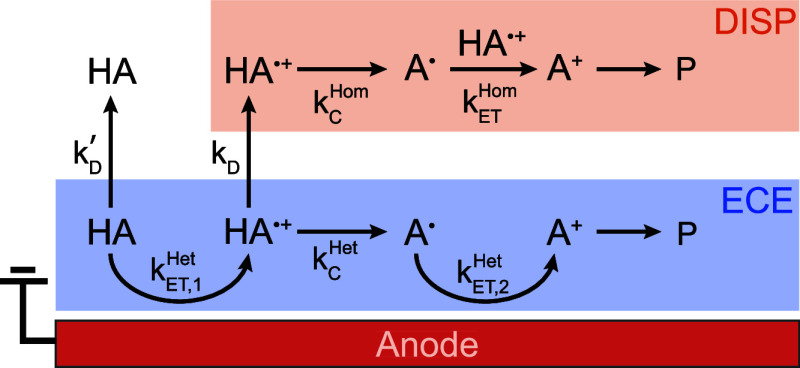
Schematic illustrating the heterogeneous ECE (blue) and homogeneous DISP (orange) mechanistic pathways for anodic two-electron oxidation of PMBA substrate denoted “HA” to the corresponding aldehyde product “P.” The diagram highlights the key intermediates and kinetic steps considered in this study.

We define two rate constants describing the (first-order) kinetics for the substrate to desorb from the working electrode surface: *k*_*D*_ *′* for the neutral PMBA substrate and *k*_*D*_ for its cation radical intermediate. As indicated in Figure 2, it is then the relative kinetics encompassed by the rate constants *k*_*D*_, *k*_*ET*_, and *k*_*C*_ that will dictate the relative branching between the ECE and DISP pathways. In this work, we will show/argue that the critical kinetic competition dictating ECE vs DISP pathways is deprotonation of the cation radical intermediate (*k*_*C*_) compared to desorption (*k*_*D*_) of the cation radical from the working electrode surface. If deprotonation of the cation radical at the electrode interface (*k*_*C*_^Het^) is slow, the cation radical will first desorb into the bulk, where it then undergoes deprotonation via a homogeneous reaction step (*k*_*C*_^Hom^). Complete reaction to the aldehyde then entails the neutral radical undergoing (homogeneous) disproportionation (eq 4) for the second electron transfer/oxidation step (*k*_ET_^Hom^). However, the neutral radical may also react via unproductive side reactions, such as HAT, radical coupling, etc.; Figure 1 depicts only the productive pathway leading to the aldehyde product.

In contrast, if deprotonation of the cation radical is fast relative to the substrate desorption from the electrode surface, the full electrochemical reaction is expected to follow the heterogeneous ECE pathway (Figure 2). This is because both the second heterogeneous electron transfer (oxidation from neutral radical to cation) and the second deprotonation (cation to aldehyde product) are expected to occur with very fast kinetics. Based on DFT calculations given in the Supporting Information, the second deprotonation step occurs barrierlessly in water (Figure S10), as demonstrated by a relaxed potential energy scan along the O–H coordinate of the cationic intermediate. We estimate that the second heterogeneous electron transfer step will be fast based on the following argument/calculations. Fast heterogeneous electron transfer for a substrate with negligible inner-sphere reorganization energy (e.g., ferrocene/ferrocenium) corresponds to a standard rate constant of *k*_ET_^0, *Het*^ ∼ 10 cm/s; this may be converted to a pseudo-first order rate constant of *k*_ET_^0, *Het*^ ∼ 10^9^ s^–1^ using estimates of the Helmholtz layer thickness (i.e., ET distance).^100–102^ Based on DFT calculations given in the Supporting Information, the second oxidation in Figure 1 has a small/moderate inner-sphere reorganization energy of ∼0.2 eV, likely leading to a smaller value for *k*_ET,2_^0,*Het*^ as compared to the value cited above. However, the major factor is the substantial overpotential for the second oxidation; DFT calculations given in the Supporting Information predict that the oxidation potential of the neutral radical intermediate (second oxidation) is ∼2 eV lower than the oxidation potential of the PMBA substrate (first oxidation). This implies that there will be a roughly 2 V overpotential driving the second heterogeneous electron transfer step when the anode is held at the working potential corresponding to the initial PMBA oxidation. The rate constant for the second heterogeneous electron transfer *k*_ET,2_^*Het*^ will thus be very large, given the exponential dependence on overpotential (i.e., Butler–Volmer kinetics).^103^

It is important to note that the considered deprotonation of the cation radical intermediate “HA^+•^” (Figure 2) corresponds to the loss of the benzylic C_α_-H proton, as depicted in Figure 1. This deprotonation is the most thermodynamically favorable since the C_α_-H is the highly acidic proton of the cation radical intermediate. We note that prior experimental studies have shown that deprotonation of the O–H alcohol group of the HA^+•^ cation radical occurs and is kinetically favored under basic conditions.^65,104^ However, under the neutral pH conditions considered here, the O–H deprotonation of HA^+•^ is not favorable and is thus not considered further.

We thus focus on the kinetic competition between deprotonation of the cation radical intermediate and its desorption from the working electrode, presenting predictions of the relevant rate constants *k*_*D*_ and *k*_*C*_^Het^ from molecular simulations. Our simulated electrochemical systems are chosen/motivated based on the experimental electrolysis reaction conditions utilized in the work of Wang et al.^28^ In the experimental study, constant current electrolysis was performed in a continuous-flow reactor using a carbon paper anode and a nickel cathode. The electrolyte consisted of a 1:1 mixture of acetonitrile and water with 0.006 M nBu_4_NBF_4_ as supporting electrolyte.^28^ Our simulations employ an idealized electrochemical cell composed of two planar graphite electrodes with pure water as the solvent and 0.4 M LiClO_4_ electrolyte (Figure 3). We simulate constant potential electrolysis rather than constant current electrolysis, as the microscopic kinetics of the latter are extremely complicated, given that microscopic rate constants will depend strongly on the changing overpotential. For instance, in addition to *k*_ET_ varying exponentially with overpotential (Butler–Volmer kinetics), our simulation results show that the substrate desorption rate constant(s) *k*_*D*_ strongly depends on the working electrode charge/overpotential. Thus, while constant current electrolysis is the common choice for preparative electrosynthesis, constant potential electrolysis (the focus in this work) is preferred/advantageous for interrogating the kinetics of elementary reaction steps, and elucidating the influence of solvent/electrolyte/double layer on the heterogeneous rate constants.

**Figure 3:**
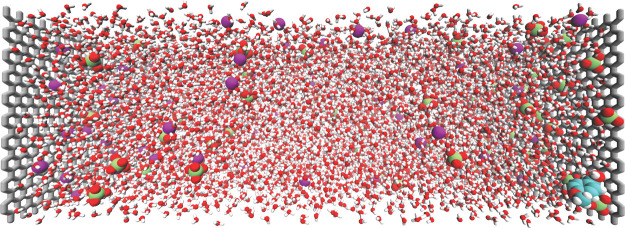
Representative snapshot of the simulation cell used in classical molecular dynamics simulations. The system consists of two graphite electrodes with surface charge mimicking working potentials with 0.4 M LiClO_4_ aqueous electrolyte.

### 3.1 Anodic Double Layer Structure and Surface Adsorption

To understand how the electrochemical environment influences substrate reaction kinetics, we first analyze the electrical double layer (EDL) structure at the graphite anode, focusing on the spatial distribution of water and LiClO_4_ components under varying electrode surface charge (σ). The EDL is a highly structured interfacial region that plays a critical role in modulating substrate–electrode interactions. Changes in its composition and organization directly influence the positioning, orientation, and adsorption/association free energies of organic substrates and their electrogenerated intermediates. We analyze the structural arrangement of water molecules and perchlorate ions at systematically varying working electrode charge (σ) to gain insight into the electrostatic/solvophobic forces that stabilize or destabilize substrate adsorption at the working electrode. Understanding the EDL structure serves as a foundation for subsequently interpreting reaction kinetics, particularly the substrate/intermediate residence times at the working electrode surface and rate constants for deprotonation reactions that occur heterogeneously at the working electrode.

The EDL structure was simulated/analyzed over the range of electrode surface charge from 0 ≤ σ ≤ 30 μC/cm^2^. We estimate that the working potential of the electrolysis corresponds to σ ∼ 14 μC/cm^2^, assuming a double-layer capacitance of *C* ∼ 10 μF/cm^2^. Details regarding our estimate of electrode charge at working potential are given in the Supporting Information, which also requires an estimate of the potential of zero charge (PZC) of the electrochemical interface in addition to the substrate oxidation potential. It is important to note that the capacitance of the electrochemical interface represents the major source of uncertainty when estimating the electrode surface charge at a given working potential.

Figure 4 shows the number density profiles of water and perchlorate anions within the anode EDL, as computed from classical molecular dynamics simulations at varying electrode surface charge. With increasing surface charge density, there is substantial restructuring of the EDL in terms of both solvent and ion structure. Peaks in water oxygen and hydrogen distributions sharpen and shift closer toward the electrode, indicating enhanced layering with a larger applied potential/charge (Figure 4a,b). With increasing electrode charge, perchlorate anions also accumulate closer to the surface, with significant density appearing within the first solvation layer (Figure 4c). The observation that perchlorate anions penetrate the inner layer of water molecules to directly contact the electrode surface differs from the classical Helmholtz model of aqueous double layers; the latter picture entails ions of closest approach (outer Helmholtz layer) separated from the electrode by the Stern water monolayer. Such differences in the observed EDL structure compared to the Helmholtz model are likely due to both the nature of electrode (carbon) and perchlorate ions; the Helmholtz model is typically postulated for mercury/solid metal electrode surfaces that are less hydrophobic than carbon, and with smaller electrolyte ions (e.g., Na^+^,Cl^–^) that have larger solvation energy.^103^ The EDL structure predicted by our simulations, with perchlorate anions readily penetrating the inner water layer to directly contact the graphite surface at moderate working potentials, is consistent with recent studies demonstrating that anion adsorption can occur at graphite–water interfaces even at or near the potential of zero charge.^105^

**Figure 4:**
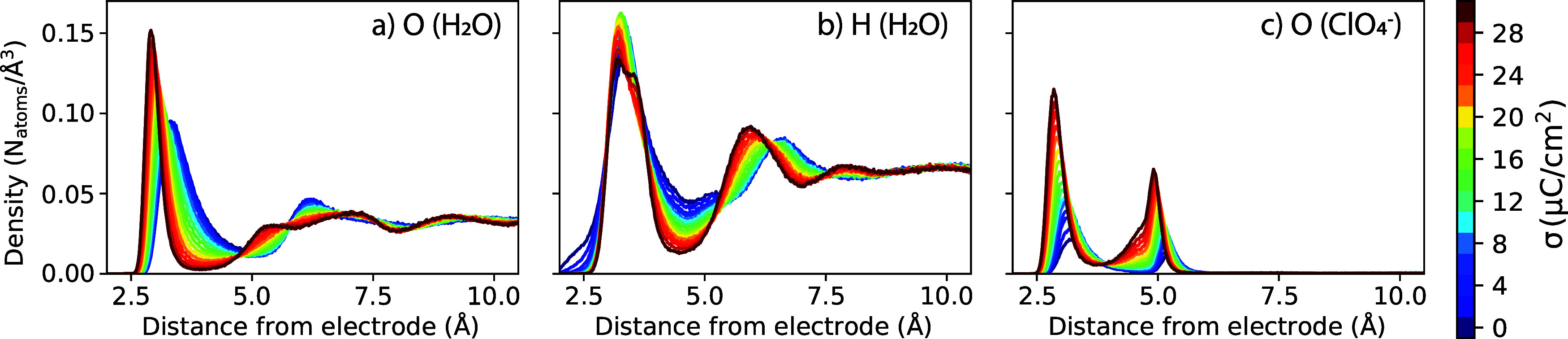
Density profiles within double layer at graphite anode with 0.4 M LiClO_4_ aqueous electrolyte. Number density is plotted for (a) oxygen atoms of water, (b) hydrogen atoms of water, and (c) oxygen atoms of perchlorate anions within the EDL. Line colors from blue to red correspond to surface charge density of the anode spanning 0 ≤ σ ≤ 30 μC/cm^2^ as given by the color bar key.

We next investigate the free energy for the PMBA substrate to associate with the electrode surface at varying working potentials/surface charge. We compute potentials of mean force (PMFs) as a function of distance between the substrate and electrode surface for both the neutral and cation radical forms of para-methoxybenzyl alcohol (PMBA). The computed PMFs, as a function of electrode surface charge, are shown in Figure 5. The distance coordinate is chosen as that between the electrode and a carbon atom on the benzyl ring (bonded to the −CH2OH group). There are clear, well-defined minima in the PMFs, which reflect preferred conformations for substrate adsorption/association with the electrode surface.

**Figure 5:**
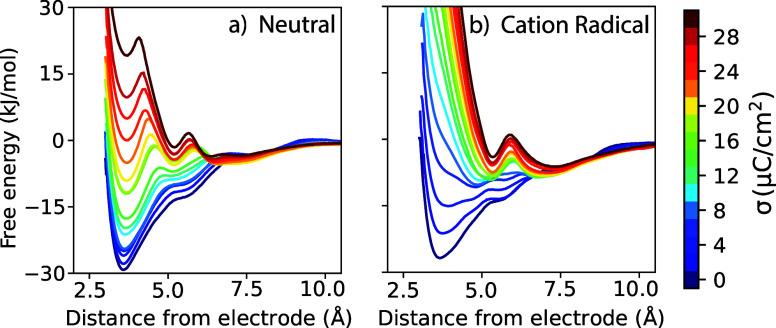
Potentials of mean force (PMFs) for (a) neutral and (b) cation radical oxidation states of the paramethoxybenzyl alcohol (PMBA) substrate as a function of distance from the graphite anode in 0.4 M LiClO_4_ aqueous electrolyte. Line colors from blue to red correspond to surface charge density of the anode spanning 0 ≤ σ ≤ 30 μC/cm^2^ as given by the color bar key.

At the potential of zero charge (σ = 0), the neutral PMBA substrate exhibits strong adsorption to the graphite electrode; the deepest/most favorable minima corresponds to the benzyl ring lying flat against the graphene surface, corresponding to a free energy minimum of approximately −30 kJ/mol at a distance of ∼3.5 Å for the substrate/electrode coordinate. This adsorption is primarily due to solvophobic forces as mediated by the aqueous electrolyte; i.e., the hydrophobic aromatic ring maximizes contact with the apolar graphene surface to minimize exposed surface area to the aqueous phase. As the surface charge on the electrode increases, this favorable interaction becomes increasingly disrupted by perchlorate anions that accumulate at the interface (Figure 4c). These anions crowd the surface and reduce the accessibility for the substrate, leading to progressive destabilization of the flat configuration. At σ > 20 μC/cm^2^, there is a change in the most favorable adsorption configuration to a second local minimum in the PMF at distance values of ∼5.5 Å for the substrate/electrode coordinate. As we will discuss below, in this configuration, the benzyl ring is tilted off the electrode surface, with the alcohol group touching the surface. The two different “flat” (distance ∼ 3.5 Å) and “tilted” (distance ∼ 5.5 Å) minima in the PMFs thus both correspond to surface adsorbed configurations with the substrate in direct contact with the electrode surface, with relative free energy substantially modulated by electrode surface charge.

Representative snapshots of the most favorable adsorption motifs are shown in Figure 6. The three configurations in panels a–c in Figure 6 correspond to the free energy minima observed at varying electrode surface charge (Figure 5a), and centered at coordinate distances of ∼3.5, ∼5.5, and ∼7.5 Å, respectively. Panel (a) depicts the “flat” motif corresponding to the minimum at ∼3.5 Å, in which the PMBA aromatic ring lies nearly parallel to the electrode, maximizing surface contact and minimizing solvent exposure. The minimum at ∼5.5 Å is depicted in panel (b), corresponding to the “tilted” motif in which the hydroxyl group is anchored at the electrode surface while the ring tilts away from the electrode into the electrolyte. This motif strikes a balance between hydrophobic and electrostatic forces, with the alcohol group able to hydrogen bond to water molecules and solvate perchlorate ions in the inner layer contacting the electrode surface. Furthermore, the benzyl ring is positioned in a “low dielectric” solvent environment as the dielectric constant of the inner water layer(s) is much reduced relative to the bulk.^98,106–111^ Lastly, panel (c) in Figure 6 corresponds to the broad/shallow free energy minima at ∼7.5 Å, in which the PMBA substrate is detached from the electrode surface slightly above the first layer of water/ions. This PMF minima has a broader orientational/translational distribution, with the attraction resulting from solvophobic/hydrophobic forces that drive the benzyl ring within/near the lower dielectric inner water layers. There is a systematic trend in the relative stability of the three configurations (a), (b), (c) with increasing electrode surface charge. The PMFs in Figure 5a indicate the substantial shift from the favorability of the “flat” configuration (panel a) at low surface charge to the “tilted” (panel b) and “desorbed” (panel c) configurations at higher surface charge.

**Figure 6:**
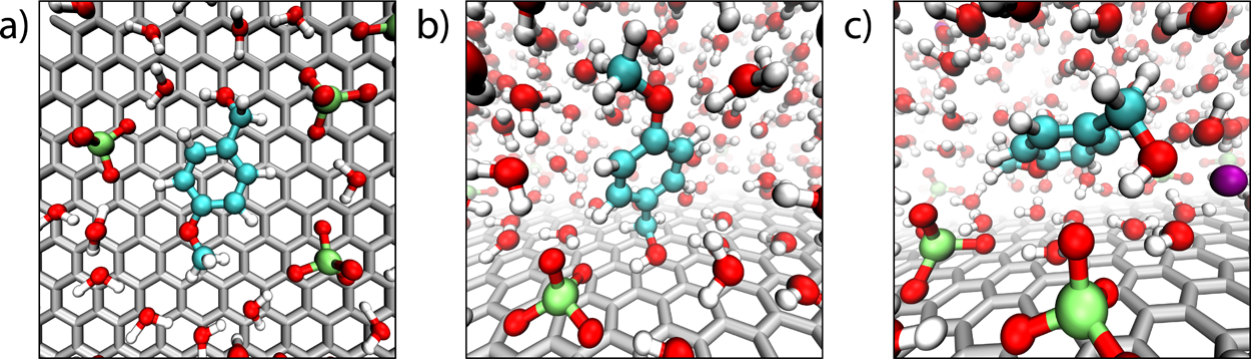
Simulation snapshots of adsorption motifs (a) flat, (b) tilted, and (c) desorbed for para-methoxybenzyl alcohol (PMBA) near the graphite anode at σ = 14 μC/cm^2^ within 0.4 M LiClO_4_ aqueous electrolyte. The motifs shown in panels (a), (b), and (c) correspond respectively to the PMF minima at ∼3.5, ∼5.5, and ∼7.5 Å in Figure 5.

Corresponding PMFs for the oxidized PMBA substrate (cation radical) are computed and are shown in Figure 5b. For the PMBA cation radical, local free energy minima corresponding to similar adsorption motifs are observed, namely the flat, tilted, and desorbed configurations depicted in panels (a), (b), and (c) of Figure 6, but their relative stabilities are modulated more strongly by the applied surface charge. At lower electrode charge, the cation radical also adopts a flat configuration as the most stable free energy minima due to solvophobic forces. However, with increasing positive surface charge, the flat motif becomes increasingly unfavorable due to electrostatic repulsion between the positively charged cation radical substrate and the anode. This drives a transition to the tilted motif (panel b of Figure 6), becoming the most stable configuration, in which the positively charged aromatic ring is displaced from the surface while the hydroxyl group remains in contact with the electrode. This configuration represents a compromise between solvophobic attraction, minimizing electrostatic repulsion with the electrode, and favorable interactions between the hydroxyl group and anions in the EDL. For the largest electrode surface charges studied, the PMF further changes such that the desorbed configuration minimum (panel c of Figure 6) becomes the global minimum, in which the cation radical is fully displaced from the electrode surface and resides just above the first water/ion layer.

The PMFs in Figure 5a,b for the neutral and oxidized PMBA substrate, respectively, illustrate how the balance of forces within the electrical double layer results in the favorable local minima for substrate configurations near the electrode. For the neutral species at low to moderate surface charge, hydrophobic forces lead to strongly favorable (∼20–30 kJ/mol) adsorption of the substrate in contact with the electrode surface. The adsorption free energy minima change substantially in relative magnitude with increasing electrode surface charge. For the cation radical species, the electrostatic repulsion with the positive electrode leads to the “tilted” configuration being most favorable for most electrode surface charge densities. However, it is important to note that even at a substantial positive electrode charge, the cation radical is not “repelled” from the electrode surface, but rather remains adsorbed/attracted to the electrode surface in the “tilted” configuration with significant free energy of attraction. The presence of well-defined free energy minima for both neutral and oxidized PMBA substrates (Figure 5) demonstrates that, due to solvophobic forces, the substrates will exhibit significant residence time near the electrode surface, which has a direct consequence for the branching between heterogeneous ECE and homogeneous DISP pathways. In the next section, we explicitly compute rate constants for the substrates/intermediates to desorb from the electrode surface at varying working charge/potential.

### 3.2 Kinetics of Substrate/Intermediate Desorption from the Electrode Surface

The PMF profiles discussed in the previous Section demonstrate that the neutral/oxidized PMBA substrate exhibits stable local conformations within the anodic electrical double layer (EDL) at varying electrode surface charge. Toward determining whether the benzyl alcohol electrolysis proceeds via the heterogeneous ECE or homogeneous DISP pathway (Figure 2), we compute the desorption rate constants *k*_*D*_ corresponding to the rate/time scale for the oxidized substrate to escape from the local free energy minima to a distance beyond 1.5 nm from the electrode surface, where the PMF is essentially flat. While the rate constant *k*_*D*_ for the *oxidized* substrate is the kinetic parameter of interest, we also compute/discuss *k*_*D*_ *′* for the *neutral* substrate, as it provides an insightful comparison. Based on the different PMFs, it is clear that these rate constants will depend sensitively on both the substrate oxidation and electrode surface charge. We henceforth use the term “desorption” to refer to this process of the substrate traversing a well-defined free energy barrier out of the inner double layer; this process is clearly distinct from bulk phase, Fickian diffusion/mass-transport. It is also clear, from the magnitude of the PMF minima and discussion above, that the terminology “desorption” does not imply “chemisorption” (which it is not), as the physical driving force is primarily solvophobic forces. Our computed values of *k*_*D*_ will subsequently be compared to the deprotonation *k*_*C*_ and electron transfer *k*_*ET*_ rate constants to determine the likely branching between heterogeneous ECE and homogeneous DISP mechanisms.

One might initially propose that rate constants *k*_*D*_ could be computed/estimated by using transition state theory (TST) in combination with the computed PMFs in Figure 5. This, however, turns out not to be the case, due to the fact that the true transition state for the desorption process is not adequately described by the single electrode/substrate distance coordinate, but rather requires a multidimensional description with additional solvation coordinate(s). The need for a collective solvation coordinate to describe the transition state has been documented in similar contexts; Geissler and co-workers demonstrated this for the case of NaCl dissociation in water,^112^ and Farahvash et al. found that desorption of CO from the Pt(100)/water interface required a solvent-based reaction coordinate to accurately define the transition state.^113^ The nature of this solvent-based collective variable that adequately describes the transition state could depend on the specific substrate, solvent, electrolyte, and electrode charge and thus must be defined on a case-by-case basis. We do not attempt to explicitly characterize this solvation coordinate in more detail here. Rather, we instead compute the desorption rate constants *k*_*D*_ from direct molecular dynamics simulations by explicitly sampling the desorption process numerous times over sufficiently long time scales.

We perform long-time scale molecular dynamics simulations in which the PMBA substrate is initially placed near the anode surface, and the MD trajectories are sufficiently long to capture multiple desorption and readsorption events, enabling direct computation of desorption time scales/kinetics. The substrate is defined to be “desorbed” from the electrode once its center of mass moves beyond 1.5 nm from the electrode surface, a distance at which the PMF becomes flat and the influence of the electrode is negligible. During these simulations and to prevent the substrate from diffusing too far into the bulk (which would prohibit sampling of the desorption process), a half-harmonic restraining potential is applied at 2 nm, serving as an upper boundary for substrate distance from the electrode surface and without interfering with dynamics near the electrode interface. The residence time τ is defined as the average duration that the substrate remains within the adsorbed region before desorbing, and the desorption rate constant is then computed as *k*_*D*_ = 1/τ.^85,86^ More detailed discussion of these simulations is given in the Supporting Information.

Simulations were conducted for both the neutral and oxidized states of PMBA for electrode surface charges of σ = 0, 6, 14, 20, and 28 μC/cm^2^. For a given surface charge, eight independent simulations were run for 250 ns to obtain sufficient statistics; these simulations were extended to 500 ns for the σ = 0 surface charge (neutral electrode), for which desorption events are less frequent. Figure 7 reports the computed residence times τ and desorption rate constants *k*_*D*_ for the neutral and oxidized PMBA substrates at different anode surface charge densities. From Figure 7, it is evident that substrate residence times vary significantly with σ and depend on the PMBA substrate charge state (neutral or oxidized). At the potential of zero charge (σ = 0), the PMBA substrate exhibits long-lived surface adsorption, with residence times of 475 ns (neutral) and 425 ns (cation radical); these long residence times are due to solvophobic forces illustrated by the deep adsorption wells in the previously discussed PMFs (Figure 5). With increasing electrode surface charge σ, the residence times are substantially reduced with enhanced desorption rate constants, particularly for the oxidized PMBA substrate (cation radical). For moderate surface charge ranging from σ = 5–10 μC/cm^2^, the residence time τ for the oxidized PMBA substrate at the anode drops to ten(s) of nanoseconds, while the residence time of the neutral PMBA substrate is hundred(s) of nanoseconds, nearly an order of magnitude larger. This difference reflects the electrostatic repulsion of the cation radical intermediate by the positively charged anode, leading to relatively shorter residence times for the oxidized substrate. For larger electrode surface charges of σ ∼ 20–28 μC/cm^2^, both species exhibit faster desorption from the anode, with residence times on the order of several nanoseconds.

**Figure 7:**
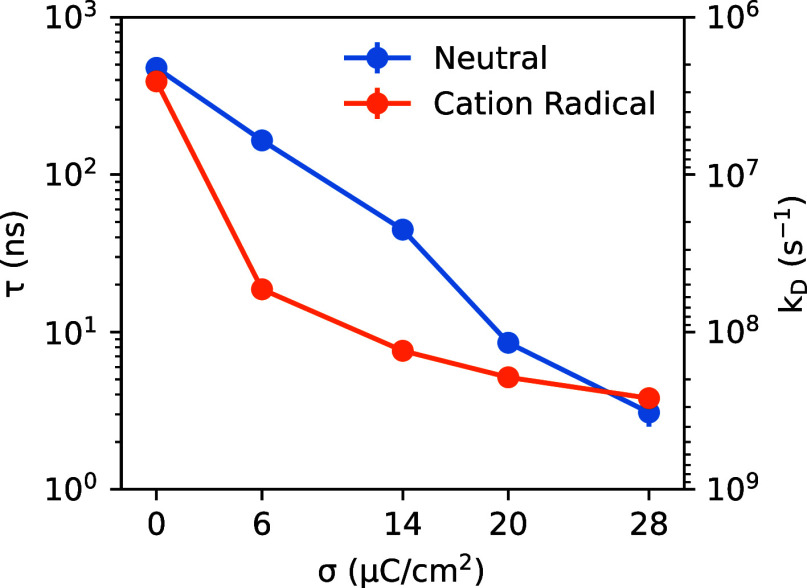
Residence times (τ) and desorption rate constants (*k*_*D*_) for neutral and oxidized PMBA substrate adsorption at the graphite anode at varying surface charge density (σ), within 0.4 M LiClO_4_ aqueous electrolyte. Although the *y*-axis is labeled *k*_*D*_, the neutral substrate data (blue) correspond to *k*_*D*_ *′* (desorption of the initial substrate), whereas the cation radical data (orange) correspond to *k*_*D*_ (desorption of the intermediate). Error bars for most data points are smaller than those of the data symbols themselves. The lines connecting the data points have no physical meaning and are simply to guide the eye.

Our predictions for the desorption rate constant *k*_*D*_, which is a key parameter in the branching between heterogeneous ECE and homogeneous DISP pathways (Figure 2), indicate that the residence time of the reactive cation radical intermediate near the anode strongly varies with the electrode surface charge, σ. While our discussion focuses primarily on *constant potential* electrolysis conditions, the implications for *constant current* electrolysis are clear; the varying/uncontrolled overpotential (e.g., surface charge) in constant current electrolysis will substantially modulate substrate/intermediate residence times and thus branching between ECE vs DISP pathways. At the estimated working electrode charge of σ = 14.6 μC/cm^2^ for constant potential electrolysis (Section 3.1), our MD simulations predict a first-order rate constant of *k*_*D*_ ∼ 10^8^ s^–1^ for the cation radical to desorb from the working anode. The key comparison for determining ECE versus DISP branching (Figure 2) is the magnitude of this desorption rate constant relative to the (pseudo first-order) heterogeneous chemical rate constant *k*_*C*_^het^ for deprotonation of the cation radical intermediate. If *k*_*C*_^het^ ≫ *k*_*D*_, then the cation radical intermediate will likely deprotonate and complete the full ECEC reaction steps (Figure 1) heterogeneously at the working anode surface before desorbing. Conversely, if *k*_*D*_ ≫ *k*_*C*_^het^, desorption of the cation radical intermediate from the anode will be faster than the chemical deprotonation step and the chemical process will likely occur homogeneously in the bulk via the DISP pathway (with disproportionation for the second electron transfer). In the next sections, we present simulation predictions of the PMBA cation radical deprotonation rate constants *k*_*C*_^het^ and *k*_*C*_^hom^, providing a more comprehensive picture of the kinetic control for the ECE and DISP pathways.

### 3.3 Kinetics of PMBA Cation Radical Deprotonation by Water

We conduct QM/MM free energy simulations as described in Section 2.3 to compute free energy profiles for the deprotonation of the PMBA cation radical intermediate, from which rate constants are estimated via transition state theory. Free energy profiles are computed for the deprotonation reaction occurring both heterogeneously at the anode surface and also homogeneously within the bulk electrolyte, providing rate constants *k*_*C*_^het^ and *k*_*C*_^hom^, respectively. Important context for our simulation predictions/results is given by experimental studies of PMBA cation radical deprotonation kinetics and, more broadly, deprotonation kinetics of the general class of alkylaromatic cation radicals. As previously mentioned, such alkylaromatic cation radicals are typically very strong acids (p*K*_a_’s ∼ −5 to −25 ^56,57^ ) yet the activation barriers for their deprotonation to either base or solvent are of moderate ∼30–60 kJ/mol magnitude, implying rate constants well below the diffusion limit.^60,61,63^ This is in sharp contrast to “normal” inorganic acids of comparable p*K*_a_, with deprotonation kinetics exhibiting small activation barriers and rate constants reaching the diffusion limit.^114–116^ For the PMBA substrate studied here, the cation radical acidity has been determined to be p*K*_a_ ≈−5.4 in acetonitrile solvent.^63^ Deprotonation rate constants for the PMBA cation radical have been measured in acetonitrile to be *k*_*C*_^hom^ = 5.7 × 10^7^ M^–1^ s^–1^ and *k*_*C*_^hom^ = 6.1 × 10^8^ M^–1^ s^–1^, with 2,6-lutidine and nitrate bases, respectively.^63^ In aqueous solutions, the pseudo-first-order rate constant for PMBA cation radical deprotonation (to water solvent) has been reported as *k*_*C*_^hom^ ∼ 1.5 × 10^4^ s^–1^ at pH 3–5, corresponding to an activation barrier of ∼50 kJ/mol.^104^

In Section 3.2, we proposed that if *k*_*D*_ ≫ *k*_*C*_^het^ for the heterogeneous desorption and deprotonation rate constants of the PMBA cation radical near the anode, then the benzylic alcohol oxidation to the aldehyde is likely to proceed via the homogeneous DISP pathway. At working potential, we predicted from MD simulations a value of *k*_*D*_ ∼ 10^8^ s^–1^ for the desorption rate constant of the PMBA cation radical from the anode surface. Thus, if we assume the *heterogeneous* and *homogeneous* deprotonation reactions exhibit similar rate constants, and taking the experimental value of *k*_*C*_^hom^ ∼ 1.5 × 10^4^ s^–1^ for PMBA cation radical deprotonation in water,^104^ we would predict that *k*_*D*_ ≫ *k*_*C*_^het^ and the DISP pathway would dominate. However, it is possible that this assumption is incorrect and that the *heterogeneous* and *homogeneous* deprotonation rates may substantially differ. The deprotonation reaction in aqueous solutions leads to the formation of a hydronium ion, the stability of which is well-known to depend sensitively on the microscopic water solvation environment.^117^ In this regard, the heterogeneous deprotonation reaction could potentially be altered by the anodic double layer structure, with its unique solvation environment characterized by strongly ordered solvent layers and an elevated ion concentration. The free energy barrier for hydronium formation depends on cooperative solvent interactions that may be disrupted by interfacial fields or local solvation changes within the double layer. To explore this question, we performed deprotonation free energy calculations within both the bulk and interfacial environment.

We predict the *homogeneous* and *heterogeneous* deprotonation rate constants *k*_*C*_^hom^ and *k*_*C*_^het^ via transition state theory (eq 5), with barriers obtained from computed free energy profiles for the deprotonation reaction. As discussed in Section 2.3, reaction free energy profiles for the PMBA cation radical deprotonation were computed with DFT-QM/MM MD simulations (at the B3LYP-D3/def2-SVP level of theory) in combination with umbrella sampling along the reaction coordinate; the reaction coordinate (Section 2.3) relates the bond distances between the proton and donor and acceptor atoms. QM/MM simulation snapshots near the transition state for PMBA cation radical deprotonation are shown in Figure 8a,b for the homo- and heterogeneous reactions, respectively. For the homogeneous reaction, the PMBA cation radical substrate and 8 solvating water molecules were included in the QM region, and the remaining solvent was treated at the MM level. As discussed in Section 2.3, the FIREs restraint was utilized to keep QM water molecules spatially localized around the cation radical reactant, and during the deprotonation reaction, the acidic proton is localized within the QM region, applying a restraint to a Voronoi-based CV that tracks the excess proton spatial location.

**Figure 8:**
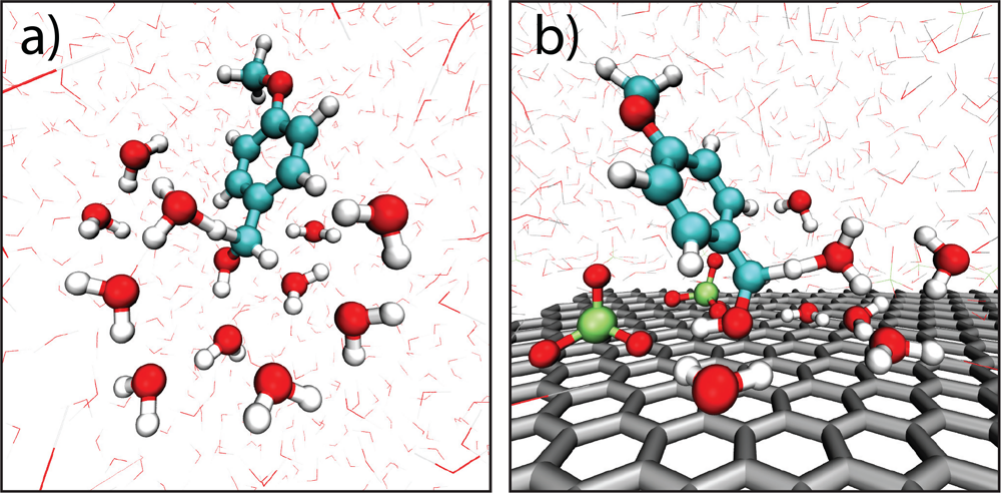
QM/MM simulation snapshots near the transition state for deprotonation of the PMBA cation radical species in (a) bulk water and (b) within the anodic double layer at σ = 14 μC/cm^2^ surface charge and 0.4 M LiClO_4_ aqueous electrolyte, for the substrate residing within the ∼5.5 Å “tilted” PMF minimum.

At the estimated electrode working charge of σ = 14 μC/cm^2^, the most probable location of the PMBA cation radical intermediate within the double layer, according to the PMFs in Figure 5b, corresponds to the “tilted” configuration at ∼5.5 Å, as depicted in the simulation snapshot of Figure 6b. We thus predict the *heterogeneous* deprotonation rate constant from QM/MM free energy profiles computed for the PMBA cation radical substrate as residing within the double layer located at this PMF minimum. Figure 8b shows a QM/MM simulation snapshot for the transition state of this heterogeneous deprotonation reaction in which the PMBA cation radical resides in the “tilted” configuration, identical to the simulation motif previously shown in Figure 6b. An identical computational approach was used as in the homogeneous deprotonation reaction, except that for the heterogeneous QM/MM free energy simulations, six water molecules were included in the QM region instead of eight, due to the lower water concentration in the double-layer environment near the electrode surface. Note that because the residence time of the PMBA cation radical intermediate within the anodic double layer at σ = 14 μC/cm^2^ working charge is on the order of nanoseconds (Figure 7), the substrate location/configuration within the double layer is well-defined during the shorter (tens of picoseconds) QM/MM umbrella sampling simulations used to compute the deprotonation free energy profile.

Figure 9 shows the free energy profiles computed for PMBA cation radical deprotonation for the *homogeneous* reaction (“Bulk”, blue curve) and for the *heterogeneous* reaction (“Interface”, orange curve). As expected from the acidity of the PMBA cation radical (p*K*_a_ ≈−5.4 ^63^), deprotonation is very thermodynamically favorable; thus, the kinetics of the reaction are the primary focus. For the homogeneous deprotonation, our QM/MM simulations predict a free energy barrier of ∼20 kJ/mol (“Bulk”, blue curve). Applying transition state theory, this gives an estimate of *k*_*C*_^hom^ ∼ 2 × 10^9^ s^–1^ for the (pseudo first-order) rate constant for deprotonation to bulk water solvent. This predicted rate constant is considerably larger than the experimentally measured rate constant of *k*_*C*_^hom^ ∼ 1.5 × 10^4^ s^–1^ for PMBA cation radical deprotonation in bulk water.^104^ In this regard, there are well-known deficiencies of DFT functionals for describing reactions of organic radical ions,^118–120^ which could lead to substantial quantitative uncertainty/error in our predicted free energy barriers. In the Supporting Information, we report corresponding QM/MM free energy profiles computed with a different basis set, namely, at the B3LYP-D3/def2-TZVPP level; the def2-TZVPP basis set leads to considerably more computationally expensive QM/MM simulations compared to the smaller def2-SVP basis set. The free energy barriers predicted from QM/MM B3LYP-D3/def2-TZVPP are 35–40 kJ/mol, substantially higher than the barriers from the QM/MM B3LYP-D3/def2-SVP simulations shown in Figure 9. The deprotonation barrier of ∼ 35–40 kJ/mol predicted from the larger basis set simulations gives a transition state theory estimate for *k*_*C*_^hom^ in closer agreement with the reported experimental value (albeit still somewhat larger). The choice of B3LYP-D3/def2-SVP as the primary level of theory for the QM/MM simulations in this work was motivated by both reduced computational cost, and previous work that demonstrated accurate prediction of cation radical reactions with DFT functionals and smaller basis sets (resulting from error cancellation presumably related to basis set incompleteness and self-interaction error in the DFT functional).^121^

**Figure 9:**
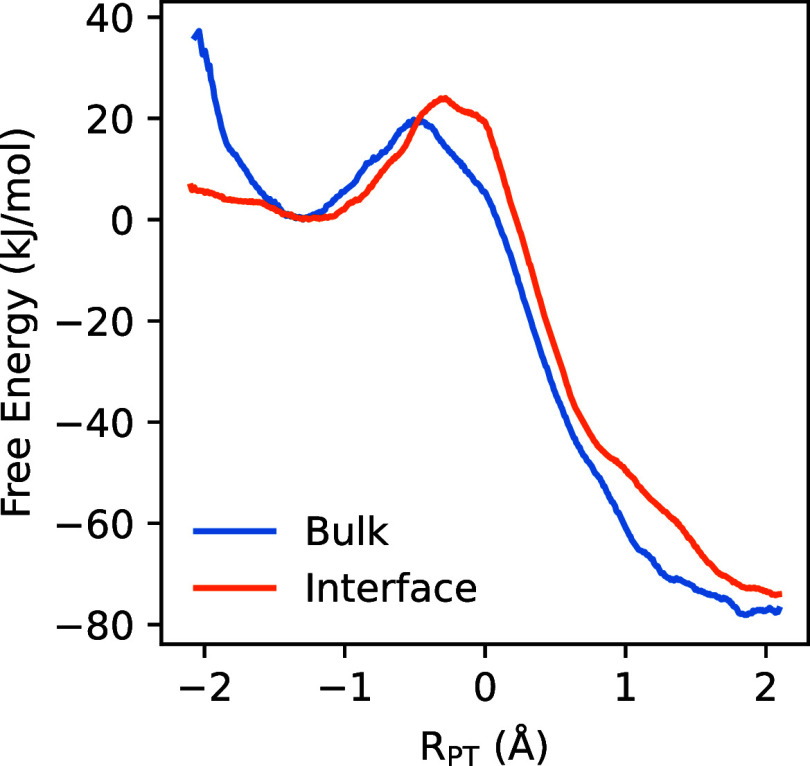
Free energy profiles for PMBA cation radical deprotonation by water within bulk water (blue curve “bulk”) and within the anodic double layer at σ = 14 μC/cm^2^ surface charge and 0.4 M LiClO_4_ aqueous electrolyte for the substrate residing within the ∼5.5 Å “tilted” PMF minimum (orange curve “interface”). Free energy profiles are computed with QM/MM at the B3LYP-D3/def2-SVP level of theory. The reaction coordinate “*R*_PT_” is defined in Section 2.3, and it relates the bond distances between the proton and donor and acceptor atoms. The transition state for deprotonation is depicted by the QM/MM simulation snapshots shown in Figure 8.

Given the uncertainties/errors associated with DFT, it is prudent to focus on *relative* (rather than absolute) differences between free energy profiles, e.g., computed in homogeneous compared to heterogeneous environments (and henceforth we focus on the B3LYP-D3/def2-SVP QM/MM predictions shown in Figure 9). Despite the very different solvation environment, the *heterogeneous* deprotonation reaction occurring within the anodic double layer exhibits a relatively similar free energy barrier of ∼24 kJ/mol (“Interface”, orange curve Figure 9) as compared to ∼20 kJ/mol for the *homogeneous* deprotonation reaction (“Bulk”, blue curve Figure 9). Seemingly, then, the different solvation environments do not lead to substantial differences between the heterogeneous *k*_*C*_^het^ and homogeneous *k*_*C*_^hom^ deprotonation rate constants, according to our QM/MM predictions. This should not be misconstrued as a general result, as is shown in Section 3.4.

The QM/MM snapshots near the transition state for both the heterogeneous and homogeneous PMBA cation radical deprotonation reactions (Figure 8) hint at the origin of the transition state barrier. Both simulation snapshots show that at the transition state, the C_α_-H bond of the benzyl cation radical acidic proton is oriented nearly perpendicular to the plane of the aromatic benzyl ring, in a “shared-proton” configuration with the acceptor water molecule. Prior experimental studies of the kinetics and product yield for alkylaromatic cation radical deprotonation reactions indeed led to the proposal of important stereoelectronic contributions to such activation barriers.^58,59,62,63^ When deprotonation occurs, one of the electrons in the C_α_-H σ bond is transferred to the aromatic pi system of the benzyl ring. Such electron transfer is facilitated by conjugation if there is overlap between the p-orbital on the C_α_ carbon with the pi-framework of the benzyl ring as deprotonation occurs,^59,63^ which is facilitated by the perpendicular alignment of the C_α_-H bond relative to the plane of the benzyl ring observed in the simulation snapshots of the transition state (Figure 8). Indeed, the geometry observed in Figure 8a suggests that the C_α_ carbon adopts more sp^2^ hybridization character at the transition state (rather than sp^3^ hybridization of the reactant state), enabling the p-orbital conjugation to the benzyl pi system required for electron transfer. We note that because such electron transfer occurs locally via orbital conjugation, it should be well described as an “adiabatic” process as captured within the Born–Oppenheimer QM/MM simulations; in lieu of such orbital conjugation/resonance, the deprotonation reaction would otherwise potentially proceed via a more complex (nonadiabatic), proton-coupled electron transfer process.^95,97,122^

To further investigate the stereoelectronic origin of the activation barrier for PMBA cation radical deprotonation, we analyzed changes in the molecular charge density along the reaction coordinate. As a proxy for the molecular charge density, we performed minimal basis iterative stockholder (MBIS) charge fitting/analysis on the substrate at various QM/MM configurations sampled along the reaction.^123^ Snapshots were extracted from the umbrella sampling simulations along the reaction coordinate, and for each snapshot, the MBIS partial atomic charges were computed. It is important to note that MBIS charges are computed from the electron density self-consistently obtained from a solution of the full QM/MM Hamiltonian, thus reflecting the electronic structure as modulated by electrostatic interactions with the surrounding environment. Charges were then summed over chemically meaningful fragments of the PMBA cation radical and QM solvent molecules to track charge localization. In addition to the charge fitting analysis, spin density distributions reflecting the radical localization were also computed from these simulation snapshots to analyze spatial changes in the radical density for the reactant, transition state, and product configurations. Representative spin density plots are provided in the Supporting Information. Because the spin density analysis leads to qualitative conclusions similar to those of the MBIS charge fitting analysis, we focus only on the discussion of the latter for brevity.

Atomic charges summed and grouped over chemical fragments as computed along the deprotonation reaction coordinate are shown in Figure 10. The *y*-axis of Figure 10 gives the summed charge “*q*_sum_” on the chemical fragment, and the *x*-axis is the reaction coordinate *R*_PT_. The individual functional groups/chemical fragments that were considered are the benzyl ring with methoxy group “*C*_6_ *H*_4_ *OCH*_3_”, the C_α_-H with *nonacidic* hydrogen “CH”, the alcohol group “OH”, and the acidic proton grouped with all solvating water molecules in the QM region “*H* ^+^ + 12*H*_2_ *O*” (note that while both C_α_ hydrogen atoms are chemically equivalent, the “acidic hydrogen” is the one chosen/labeled for the biased sampling, deprotonation reaction). The variation of charges on these functional/chemical groups along the reaction coordinate clearly illustrates the shift of positive charge from the aromatic ring to a newly formed hydronium ion at the proton-accepting water molecule. Before the transition state (which is labeled by a vertical dashed line on the graph), the excess positive charge is mostly localized on the benzyl ring (blue, “*C*_6_ *H*_4_ *OCH*_3_” group), remaining nearly constant until the reaction reaches the transition state. After crossing the transition state, there is a continuous transfer of positive charge from the benzyl ring to the solvated acidic proton (red, “*H* ^+^ + 12*H*_2_ *O*” group), clearly visualized in Figure 10 from correlated changes in the blue and red curves. This positive charge transfer corresponds to one of the electrons in the C_α_-H σ bond being transferred to the aromatic pi system of the benzyl ring, as correlated with the deprotonation event. As shown in the Supporting Information, accompanying charge transfer is a transfer in spin density, in which the spin density becomes much more localized on the C_α_ carbon atom following proton abstraction (Figure S6). The charge analysis given in Figure 10 is from the PMBA cation radical deprotonation in bulk water, and a similar profile/interpretation exists for the heterogeneous deprotonation reaction within the double layer (Supporting Information).

**Figure 10:**
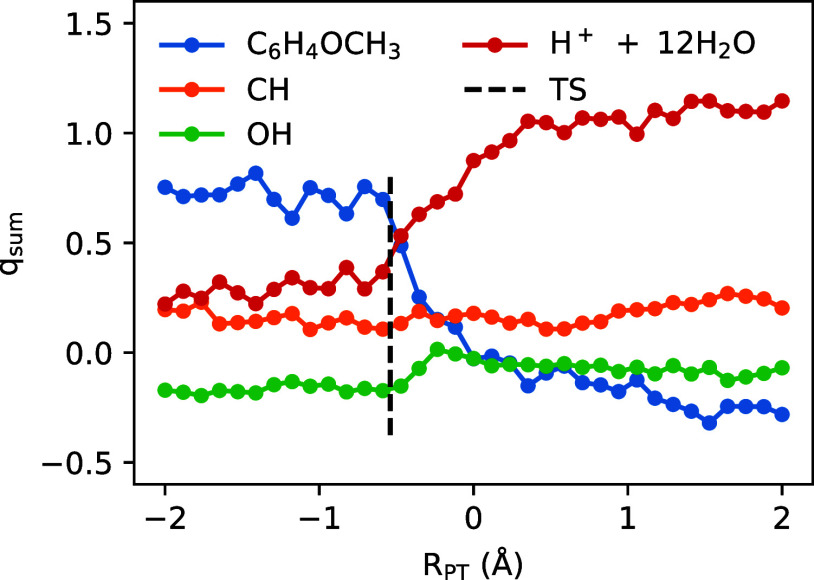
MBIS atomic charges fit for the PMBA cation radical from QM/MM simulation snapshots along the proton transfer coordinate (*R*_PT_) for the homogeneous deprotonation reaction in bulk water. Charges are fit from electron density computed from the full QM/MM Hamiltonian at the B3LYP-D3/def2-SVP level. Individual atomic charges are summed and grouped according to the labeled functional groups. *R*_PT_ = −0.54 corresponds to the transition state and is labeled with a vertical black dashed line.

The takeaway from our computed PMBA cation radical deprotonation free energy profiles and corresponding analysis is that the kinetics of this chemical step is likely a key factor dictating the ECE versus DISP mechanistic branching of the electrolysis reaction. The moderate activation barrier for cation radical deprotonation results in large part from stereoelectronic effects, in agreement with findings from previous experimental work investigating alkylaromatic cation radical deprotonation kinetics.^58,59,62,63^ Any substituent effect or change in reaction conditions (e.g., presence of base) that would lower the activation barrier for cation radical deprotonation may thus lead to promotion of the heterogeneous ECE pathway (with faster deprotonation) and possible modulation of electrolysis product yield/selectivity (due to side reactions within DISP pathway). We explore this hypothesis in the next section, considering the effect of the added base on the deprotonation barrier/rate.

### 3.4 Kinetics of PMBA Cation Radical Deprotonation by Acetate: Role of the Electrical Double Layer

If the ECE pathway is potentially desirable for mitigating radical side reactions, it may then seem intuitive to add a stronger base (compared to water) to facilitate deprotonation of the PMBA cation radical. Indeed, in the work of Wang et al. with very high reported benzyl aldehyde product yields from direct electrolysis, the proposed mechanism depicted corresponding cation radical deprotonation by hydroxide base rather than water (although it is unclear how hydroxide would accumulate at the anode for the given reaction conditions).^28^ However, given the stereoelectronic origin of the deprotonation barrier, it is uncertain to what extent the addition of base would accelerate the deprotonation kinetics; indeed, for certain alkylaromatic cation radicals, experiments have demonstrated the lack of correlation between thermodynamic driving force (p*K*_a_) with deprotonation rates.^60^ In this section, we investigate corresponding PMBA cation radical deprotonation within a sodium acetate electrolyte. As described in Section 2.1, an electrochemical cell was constructed analogous to before, except with a 0.4 M NaOAc electrolyte instead of the 0.4 LiClO_4_ electrolyte considered in Sections 3.1–3.3. Similar simulation results/discussion to those given previously are presented here for the 0.4 M NaOAc electrolyte system.

There is experimental precedent for similar electrolysis of benzyl ethers with NaOAc electrolyte (within methanol solvent).^25^ Garwood et al. conducted electrolysis of *p*-methoxy benzyl methyl ether in NaOAc/methanol utilizing a platinum anode, obtaining a relatively good yield (72%) of the benzyl aldehyde product. The reaction is assumed to proceed via a similar mechanism as that shown in Figure 1, except the benzyl cation (formed from loss of 2e^–^, H^+^) is attacked by methanol/OAc to form either an acetal or hemiacetal acetate, which is then hydrolyzed during workup to give the aldehyde product.^25^ This difference is unimportant for the present discussion, since our focus is on the initial benzyl cation radical deprotonation step, which proceeds via an analogous mechanism in either case. We note that there may be some practical concern with regard to the oxidation potential of the acetate anion being quite similar to that of the PMBA substrate;^26,124^ thus, the oxidative stability of the NaOAc electrolyte may be very sensitive to local pH (as reported for the Kolbe electrolysis^15^) and likely depends on experimental conditions. Given the experimental precedent for electrolysis within NaOAc/MeOH,^25^ we do not discuss this issue further here.

Our goal here is to investigate whether the acetate anions may promote lower barriers for PMBA cation radical deprotonation and how this reaction may be influenced by the structured electrical double layer environment. In this regard, it is important to note that the anodic double layer environment is expected to be highly concentrated with acetate anions under working conditions, substantially differing from the 0.4 M NaOAc concentration in the bulk. We start by discussing the double-layer structure and potentials of mean force (PMFs) for substrate/electrode adsorption, within the aqueous 0.4 M NaOAc electrolyte with anode at working potential (σ = 14 μC/cm^2^). Figure 11a shows the PMF for PMBA cation radical association with the carbon anode at the working charge, and Figure 11b shows the corresponding density profiles of various chemical groups within the anodic double layer. The PMF for substrate/electrode adsorption in Figure 11a appears quite similar to that previously computed/discussed for the 0.4 M LiClO_4_ system in Figure 5b at a similar working charge (σ = 14 μC/cm^2^). We note that the axis scales are chosen differently in Figure 11a and Figure 5b for visual clarity. The two minima in the PMF profile of Figure 11a at ∼5.5 and ∼7 Å correspond to similar motifs as shown in Figure 6b,c, respectively, in which the PMBA cation radical is either “tilted” with hydroxyl group anchored to electrode surface or the substrate is detached from the electrode surface residing slightly above the first solvent/ion layer. There is a subtle difference in the PMFs within the 0.4 M LiClO_4_ and 0.4 M NaOAc aqueous electrolytes, in that the “tilted” motif (5.5 Å minimum) is slightly destabilized relative to the “detached” motif (7 Å minimum) within the NaOAc electrolyte (Figure 11a).

**Figure 11:**
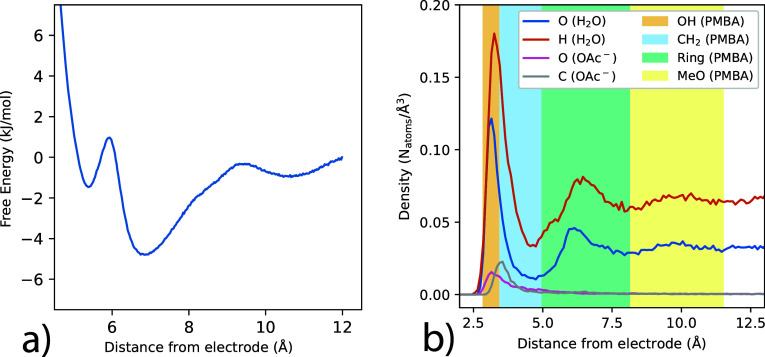
(a) Potential of mean force (PMF) for PMBA cation radical substrate as a function of distance from the graphite anode with surface charge σ = 14 μC/cm^2^ and with 0.4 M NaOAc electrolyte. (b) Density profiles within the anodic double layer of the same system; functional group distribution of PMBA is indicated by shaded color regions, as computed for PMBA in the “tilted” configuration corresponding to the first minimum observed in the PMF.

Figure 11b shows the anodic double layer density profiles at σ = 14 μC/cm^2^ working charge. Full density profiles showing the density of all species and PMBA functional groups are given in Figure S8, while in Figure 11b and the most probable location of the PMBA cation radical is simply “color coded” by functional group for visual clarity. There is a substantial density of acetate anions at the anode surface, with the interfacial anion concentration enhanced by more than an order of magnitude compared to the bulk electrolyte (Figure S8). Figure S8 shows that Na^+^ cations are almost entirely excluded from the anodic double layer. The color coded, vertical rectangular regions denote the most probable location of the PMBA functional groups when the cation radical resides in the “tilted” configuration, i.e., the 5.5 Å minimum of the PMF in Figure 11a. Notably, both the −OH alcohol group (dark tan rectangle) and the C_α_-H_2_ functional group of PMBA are positioned close to the layer of acetate anions situated against the electrode. This implies that although deprotonation of PMBA cation radical by acetate would formally be expected to follow second-order kinetics, an “encounter pair” of the cation radical and acetate ion(s) is highly likely to be preformed when the substrate resides within the high ion content double layer. Indeed, from the simulation trajectory, it is common to observe the PMBA cation radical substrate in a configuration with its alcohol group hydrogen bonding with an acetate anion residing in the double layer (as consistent with the density distribution in Figure 11b.

There are other important features related to the PMBA cation radical substrate’s conformation within the double-layer solvation environment. The probable location of the benzyl ring is indicated by the green color-coded region; there is moderate orientational freedom for the alignment of the ring relative to the electrode, as indicated by the broad, light-yellow region depicting the probable location of the methoxy group. It is observed that the benzyl ring of the PMBA substrate mostly resides within the second and third water layers near the anode surface, as indicated by the oxygen (blue) and hydrogen (orange) water density peaks in the density profile. Notably, it is well-known that “inner” water layers within the double layer exhibit substantially reduced dielectric constant relative to the bulk.^98,106–111^ This means that the positively charged, benzyl ring of the PMBA cation radical is relatively poorly screened by the inner layer water molecules and likely exhibits enhanced Coulombic attraction with acetate anions that are concentrated within the inner layer adjacent to the anode surface. This ion pairing is another driving force for forming “encounter pairs” of the cation radical and acetate ion(s) that may precede the deprotonation reaction. The acetate anions form a negatively charged anion layer interspersed with water (Figure 11b) adjacent to the positive electrode surface, which separates the positively charged benzyl ring from the positive electrode surface, mitigating Coulomb repulsion. Within the low-dielectric, inner water/solvent layers, these ion–ion interactions are of pronounced significance.

Motivated by the observation of preformed “encounter pairs” between the PMBA cation radical and acetate ion(s) within the double layer, we conducted QM/MM simulations to compute the free energy profile for proton transfer from the cation radical to a coordinated acetate anion. Details of the QM/MM free energy simulations are given in Section 2.3; Simulation details are similar to those reported in Section 3.3, but here the “QM region” includes only the PMBA cation radical substrate and acetate base (and no water molecules), given that the (initial) deprotonation product is acetic acid rather than hydronium ion. Deprotonation reaction free energies were computed in two different environments: “Bulk,” corresponding to proton transfer between the cation radical substrate and acetate in bulk water; “Interface,” corresponding to the PMBA cation radical residing within the first minimum of the PMF (Figure 11) within the anodic double layer. Free energies for the homogeneous and heterogeneous deprotonation reactions from the QM/MM simulations are shown in Figure 12a. Consistent with Section 3.3, results are shown from QM/MM simulations conducted at the B3LYP-D3/def2-SVP level of theory. As discussed in Section 3.3, there is moderate basis set dependence of the quantitative predictions with such benchmarks given in . We focus our discussion on relative trends in the predictions, which are robust to the chosen level of theory ().

**Figure 12:**
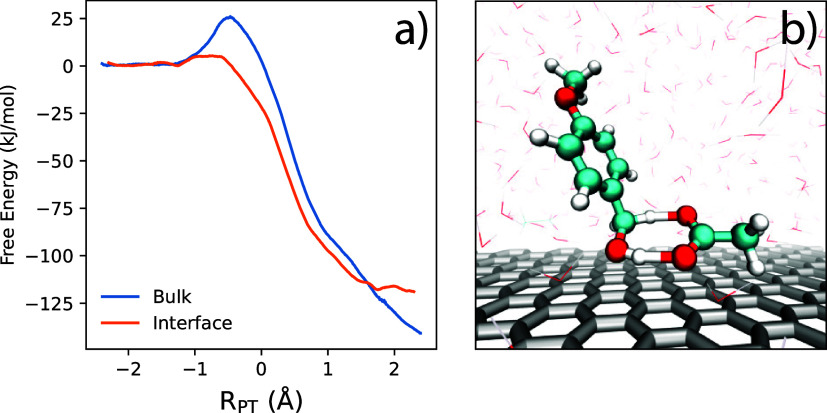
(a) Free energy profiles for PMBA cation radical deprotonation by acetate anion in bulk water (blue curve “bulk”) and within the anodic double layer at σ = 14 μC/cm^2^ surface charge and 0.4 M NaOAc electrolyte, for the substrate residing within the ∼5.5 Å “tilted” PMF minimum (orange curve “interface”). Free energy profiles are computed with QM/MM at the B3LYP-D3/def2-SVP level of theory. (b) QM/MM simulation snapshot of the transition state for PMBA cation radical deprotonation by acetate within the anodic double layer environment (“interface”), indicating hydrogen bonding between the acetate and both the *C*_α_–H and the hydroxyl group of PMBA.

*The free energy profiles in* Figure 12 *a show a compelling prediction that the barrier for proton transfer of PMBA cation radical to acetate anion is markedly dependent on the interfacial reaction environment.* For the homogeneous deprotonation reaction in bulk water, Figure 12a indicates a free energy barrier of ∼26 kJ/mol, which is largely comparable to the barrier predicted for PMBA cation radical deprotonation to water base in Figure 9. Since acetate is a stronger base than water, this implies that while the basicity clearly dictates the thermodynamics of the reaction (Figure 12a vs Figure 9), it does not necessarily alter the kinetic barrier to deprotonation. This is consistent with the experimental finding by Baciocchi et al. that deprotonation rate constants for the PMBA cation radical with 2,6-lutidine and nitrate bases (in acetonitrile solvent) did not correlate with the relative basicity (i.e., larger rate constant with nitrate compared to 2,6-lutidine).^63^ The very interesting effect that we observe in Figure 12a is that the deprotonation barrier is substantially modulated within the anodic double layer compared to the bulk solvent environment. When the reaction occurs near the electrode surface, with the PMBA cation radical situated within the first minimum of the PMF (∼5.5 Å), the barrier is reduced to approximately 5 kJ/mol.

The prediction of a substantially reduced activation barrier for PMBA cation radical deprotonation to acetate at the anode surface implies a mechanistic shift toward the heterogeneous ECE pathway for the electrolysis reaction within the NaOAc electrolyte. As shown in Section 3.3, the moderate activation barriers computed for the corresponding reaction (to water base) within aqueous LiClO_4_ electrolyte suggested that this deprotonation step would likely be rate-limiting within the heterogeneous ECE pathway. Our prediction that this deprotonation step is nearly barrierless within the NaOAc double layer (“Interface,” Figure 12a) suggests a significant modulation in the kinetic control of the ECE pathway, with deprotonation no longer rate-limiting. There are, of course, errors/uncertainties in the DFT level of theory that affect the absolute free energy barriers predicted in Figure 12a. Figure S5 shows independent reaction free energy predictions for deprotonation within the NaOAc double layer (“Interface”) computed from QM/MM simulations with larger def2-TZVPP basis set (B3LYP-D3/def2-TZVPP) compared to predictions in Figure 12a computed at the B3LYP-D3/def2-SVP level; the predicted barrier changes from 5 to 13 kJ/mol going from B3LYP-D3/def2-SVP to B3LYP-D3/def2-TZVPP level of theory. However, relative trends in predicted barrier heights are robust and largely consistent when compared across a similar level of theory (Figure S5). We note that while there are some concerns about statistical sampling of the atomistic double layer environment during the relatively short (∼10s ps) QM/MM MD simulations, the consistent trends predicted by independent QM/MM simulations at both B3LYP-D3/def2-SVP and B3LYP-D3/def2-TZVPP levels of theory give confidence in the simulation predictions.

The origin of the substantially reduced deprotonation barrier within the NaOAc double layer results from stereoelectronic effects that were discussed in Section 3.3. Figure 12b shows a representative snapshot of the reaction transition state from the QM/MM simulations. From this snapshot, it is observed that the acetate anion forms hydrogen bonds simultaneously to the acidic C_α_–H and −OH alcohol hydrogen atoms of the PMBA cation radical at this transition state configuration. The propensity for hydrogen bonding between the PMBA cation radical alcohol group and acetate ions when the substrate resides within the PMF minima of the anodic double layer (Figure 11b) means there is a high probability that such an “encounter pair” is preformed. As depicted in the Figure 12b snapshot, the hydrogen-bonded acetate anion is closely positioned to the C_α_–H group to facilitate the deprotonation reaction. As the proton transfer proceeds, this dual hydrogen bonding configuration is observed throughout the transition state region along the reaction coordinate until the proton is fully transferred and the product acetic acid rotates into a different hydrogen bonding configuration (Supporting Information). The facile deprotonation kinetics within the NaOAc double layer (Figure 12a) as predicted within our QM/MM simulations is thus likely dependent on the bidentate nature of the acetate anion; at this point, we could only speculate on whether related anions (carboxylates, oxalates, sulfonates) may interact similarly.

Section 3.3 discussed a stereoelectronic rationalization of the activation barrier, based on the necessity for the C_α_–H bond of the acidic proton to orient perpendicular to the plane of the benzyl ring to facilitate electron transfer into the pi system accompanying deprotonation. Indeed, the simulation snapshot in Figure 12b shows the C_α_–H bond positioned in such an orientation for the transition state of PMBA cation radical deprotonation to acetate within the double layer. To better rationalize the predicted deprotonation activation barrier, we compute the average dihedral angle ⟨ϕ_HCCC_⟩ encompassing the H–C_α_ atoms and the nearest two carbon atoms of the benzyl ring along the deprotonation reaction coordinate. The value of ϕ_HCCC_ ∼ 90° corresponds to the C_α_–H bond oriented perpendicular to the plane of the benzyl ring. Figure 13 shows computed values of ⟨ϕ_HCCC_⟩ along the reaction coordinate for the low-barrier (∼5 kJ/mol) deprotonation of PMBA cation radical to acetate anion within the double layer (“Interface,” Figure 12a). As a reference, we show a similar analysis for the deprotonation reaction in bulk water (with a water molecule as the base) that was discussed/presented in Figure 9. Molecular snapshots are also shown in Figure 13 that precisely illustrate the definition of the dihedral angle, ϕ_HCCC_.

**Figure 13:**
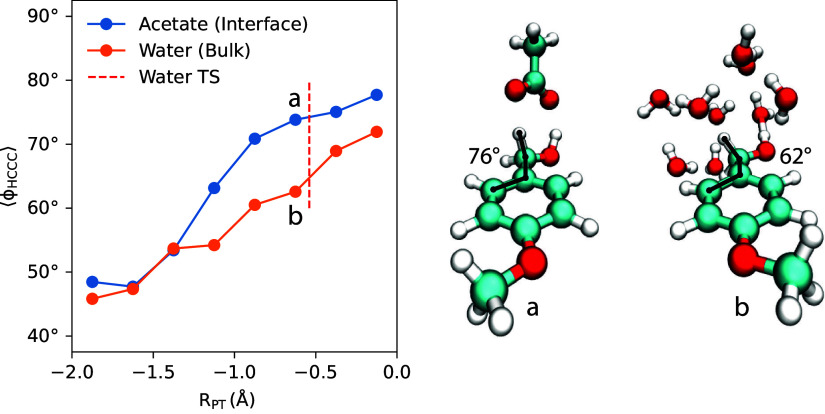
(Left) Average dihedral angle ⟨ϕ_HCCC_⟩ as a function of reaction coordinate *R*_PT_, for deprotonation of PMBA cation radical by acetate base within anodic double layer at σ = 14 μC/cm^2^ surface charge and 0.4 M NaOAc electrolyte at the “tilted” PMF minimum (“interface” blue curve) and by water base in bulk water solution (“bulk” orange curve). The vertical dashed red line marks the transition state for deprotonation by water. Points labeled “a” and “b” correspond to representative geometries shown on the right. (Right) Snapshots from the acetate (a) and water (b) deprotonation trajectories at *R*_PT_ = −0.54, with dihedral angles (76° and 62°, respectively) highlighted with black lines.

Figure 13 indicates a clear difference in the ϕ_HCCC_ dihedral distribution along the reaction coordinate for the two different deprotonation reaction conditions. For the deprotonation of PMBA cation radical to acetate anion within the double layer (“Acetate (Interface)”), ϕ_HCCC_ dihedral values are relatively larger, closer to the ϕ_HCCC_ ∼ 90° orientation that corresponds to the C_α_–H bond oriented perpendicular to the plane of the benzyl ring. Note that at the transition state for PMBA cation radical deprotonation to water (orange curve, “Water (Bulk)”), the average dihedral angle is ⟨ϕ_HCCC_⟩ ∼ 60–65° such that the C_α_–H bond does not yet reside in perfect perpendicular orientation relative to the ring. In contrast, for deprotonation to acetate anion within the double layer, this ⟨ϕ_HCCC_⟩ ∼ 60–65° alignment threshold occurs much earlier along the reaction coordinate, so that the majority of configurations on the “reactant” side exhibit at least this extent of C_α_–H bond perpendicular orientation to the ring (except for the very left side of the reaction coordinate). This implies that complexation with the acetate anion within the double-layer environment promotes PMBA cation radical configurations that are predisposed to the geometric constraints required for deprotonation. The substantially reduced activation barrier (Figure 12a) is thus of stereoelectronic origin, with the necessary C_α_–H orbital alignment/overlap with the benzylic pi system achieved well before the transition state region of the reaction coordinate.

What aspect of the anodic double layer structure and complexation between acetate and the PMBA cation radical leads to the configurational distribution reported in Figure 13, which results in a substantially reduced deprotonation barrier? As visualized in simulation snapshots of Figures 12b and 13, clearly the bidentate nature of the acetate anion plays a role, with dual hydrogen bonding between acetate and the C_α_–H and alcohol O–H hydrogen atoms mediating the ϕ_HCCC_ dihedral distribution, and promoting the orientation of the C_α_–H bond perpendicular to the benzyl ring. However, there is an additional unique role of the double-layer environment, given that the reduction in activation barrier occurs only within the double layer and not in the bulk solution (Figure 12a). The most pertinent features of the double layer, in this regard, are the strongly modulated electrostatics and steric constraints of the electrode/solution interface. The complexation and strong Coulombic interaction between acetate and the PMBA cation radical are pronounced in the double layer, given the reduced dielectric screening of interfacial solvent and the role of acetate in “shielding” the cation radical from the positively charged electrode surface. Coulombic forces likely promote the orientation of the positively charged benzylic ring toward the carboxylate group, as observed in the snapshot in Figure 12b. Furthermore, within the double layer and at the anode surface, the PMBA cation radical resides in the “tilted” motif (first PMF minimum, Figure 11a) with reduced configurational freedom, which leads to more ordered and structured complexation with the acetate anion.

As a final analysis, we conduct MBIS atomic charge analysis for the low-barrier, “Interface” deprotonation reaction to acetate within the double layer. This charge analysis is shown in Figure 14, and is similar to the previous analysis presented in Figure 10, with charge groups by similar chemical/functional groups. The only difference here is that the acidic proton is grouped with the acetate base as “*H* ^+^ + *OAc* ^–^,” rather than with water clusters in the prior analysis of Figure 10. The charge analysis in Figure 14 reveals that the transfer of charge between the benzylic ring and acidic proton/acetate begins to occur well before the transition state along the reaction coordinate (note the “transition state” here is loosely defined, given the nearly barrierless free energy profile). This contrasts sharply with the previous analysis of Figure 10 for PMBA cation radical deprotonation by water, in which the charge transfer occurs only after the transition state region. This charge analysis is fully consistent with the ϕ_HCCC_ dihedral distribution of Figure 13, since the C_α_–H orbital alignment with the benzylic pi system is required to facilitate the charge transfer.

**Figure 14:**
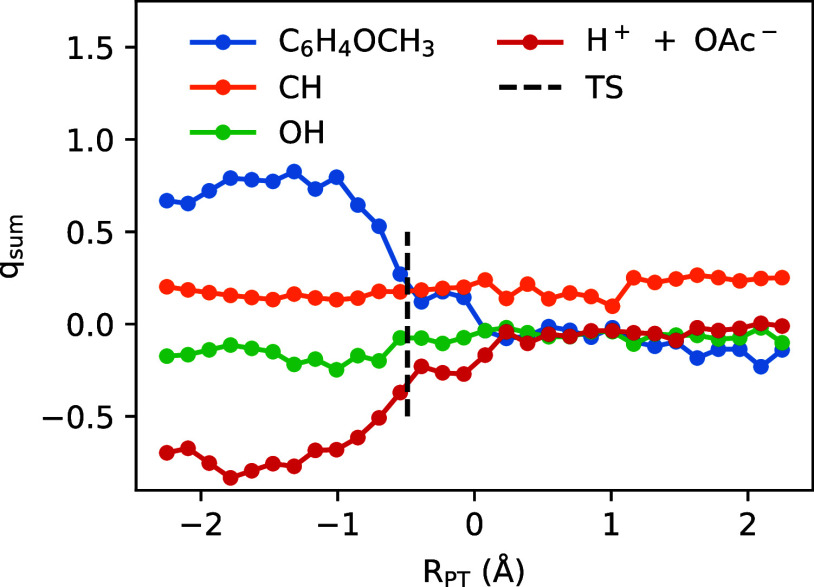
MBIS atomic charges fit for the PMBA cation radical from QM/MM simulation snapshots along the proton transfer coordinate (*R*_PT_) for the deprotonation to acetate base within the anodic double layer at σ = 14 μC/cm^2^ surface charge and 0.4 M NaOAc electrolyte, for the substrate residing within the ∼5.5 Å “tilted” PMF minimum. Charges are fit from electron density computed from the full QM/MM Hamiltonian at B3LYP-D3/def2-SVP level. Individual atomic charges are summed/grouped according to the labeled functional groups. *R*_PT_ = −0.49 corresponds to the transition state and is labeled with a vertical black dashed line.

## 4 Discussion and Conclusions

Pons and co-workers previously reported a detailed experimental kinetic study providing rate constants for ECE and DISP mechanisms of a related methylbenzene electrolysis as investigated by means of electrochemical and spectroelectrochemical methods.^54^ These authors, and others,^22,46^ have discussed clear limitations for determining rate constants and distinguishing between ECE and DISP pathways via conventional electroanalytical methods. Following an involved analysis of the spectroelectrochemical data and several kinetic assumptions, Pons and co-workers determined that electrolysis of methylbenzenes predominantly followed the DISP pathway.^54^ However, there were notable differences between their experiment and the benzyl alcohol electrolysis studied here. First, rate constants for the kinetically limiting, cation radical deprotonation step were reported as *k*_*C*_ ∼ 10^2^–10^3^s^–1^ (pseudo-first order), which is one to two orders of magnitude lower than the experimentally reported rate constant for PMBA cation radical deprotonation,^104^ and orders of magnitude lower compared to our computationally predicted deprotonation rate constants. Second, the reaction conditions were different, with the prior electrolysis utilizing a platinum working anode and acetonitrile solvent;^54^ this could clearly lead to differences in the deprotonation rate and residence time of electrogenerated intermediates at the working electrode surface.

The desorption rate constant *k*_D_, which reflects the residence time of the electrogenerated cation radical at the working electrode, is a critical kinetic parameter dictating ECE vs DISP pathway branching. In lieu of chemisorption and as dictated predominantly by solvophobic forces, this rate constant is expected to span magnitudes of *k*_D_ ∼ 10^6^–10^9^s^–1^ for substrates and reaction conditions similar to those studied here. It is difficult to experimentally determine rate constants of this magnitude via standard electroanalytical methods,^23^ and direct molecular dynamics simulations (analogous to those performed here) are thus very useful in this regard. Given such values of *k*_D_ and the empirical data on activation barriers for deprotonation of alkyl aromatic cation radicals (typically ∼ 30–60 kJ/mol),^60,61,63^ one may expect deprotonation to generally be rate-limiting, and forcing the DISP pathway rather than ECE. The ECE pathway would obviously be more likely for substrates with lower deprotonation barriers of their cation radical intermediates. ECE would be promoted by longer cation radical residence times at the working electrode (smaller desorption rate constants *k*_D_). Such residence times will be influenced by working electrode charge (determined by both substrate oxidation potential and capacitance of electrochemical interface), solvophobic forces mediated by solvent/electrolyte, and any direct chemical interaction with electrode material and/or electrostatic interactions with anions in the double layer.

We have presented a compelling computational prediction that activation energies for deprotonation may be substantially modulated for heterogeneous reactions occurring at the electrode interface within the double layer. For PMBA electrolysis within the NaOAc aqueous electrolyte, “encounter pairs” between the PMBA cation radical and acetate anions are probable, given the orientation of the PMBA alcohol group at the electrode surface and highly enhanced concentration of acetate anions within the anodic double layer. Furthermore, the combined electrostatics/sterics imposed by the electrode interface biases configurations of the cation radical/acetate complex toward a more perpendicular alignment of the C_α_–H bond relative to the plane of the benzyl ring. There is a direct stereoelectronic influence on the activation barrier, as such alignment facilitates electron transfer from the C_α_–H bond to the pi-system of the benzyl ring during the deprotonation reaction. There are substantial implications for the mechanism of benzylic alcohol electrolysis if this computational prediction is valid. Within the aqueous LiClO_4_ electrolyte, ECE would likely not be the majority pathway (rather DISP would occur to an appreciable or dominant extent) due to the rate-limiting cation radical deprotonation as compared to residence time at the working electrode interface. Within aqueous NaOAc electrolyte, however, the predicted (significant) barrier reduction implies a shift to the ECE pathway, if deprotonation rates are faster than cation radical desorption from the electrode surface. Such a change in the branching between ECE vs DISP pathways would likely alter observed product yield/selectivity, given the susceptibility of the benzyl radical intermediate to competing side reactions. We note that a limitation of our computations is neglect of the local acidity near the anode that is built up during the oxidation reaction, which is a general issue in anodic electrosynthesis that can effect reaction yield.^15,125^

We wish to conclude by noting a connection with the relevant and compelling prediction made by Eberson and Nyberg over 50 years ago in their beautifully insightful Accounts of Chemical Research article.^33^ In the context of anodic aromatic substitution reactions, these authors noted the importance of a “π-type adsorption complex between an aromatic hydrocarbon and the electrode surface” for which “the electrode might sterically control the anodic process.”^33^ To demonstrate this effect, the authors performed anodic electrolysis on 2-*tert*-butylindan and 1-*tert*-butylacenaphthene substrates, investigating acetoxylation of the substrate presumably via an ECEC mechanism. The finding was a pronounced cis/trans enhanced product ratio, with nucleophilic attack of acetate to form the cis conformer promoted by steric effects based on aromatic ring stacking against the electrode surface. Our computational predictions of modulated PMBA cation radical deprotonation kinetics within the aqueous NaOAc electrolyte double layer similarly illustrate how heterogeneous reaction rate constants can be modulated by the double layer environment and/or electrode sterics. Modern computer simulation methods such as DFT-QM/MM have now reached maturity to enable the investigation of such “electrode steric control” on electrochemical reactivity and kinetics that was insightfully postulated and demonstrated by Eberson and Nyberg decades ago.

## Supplementary Material





## Notes

Bulk electrolyte concentration analysis for simulated systems at varying electrode surface charge; atomic charges fit for neutral and cation radical states of the para-methoxybenzyl alcohol (PMBA) substrate; estimation of electrode surface charge densities at working electrolysis conditions; simulation details for substrate desorption rate constant calculations; QM/MM umbrella sampling protocols and restraint schemes for proton transfer reactions; Lennard–Jones parameter tuning and RDF benchmarking for proton transfer compatibility; Voronoi polyhedra-based CVs for excess proton localization; time-resolved convergence of umbrella sampling PMFs; basis set benchmarking of deprotonation barriers (def2-SVP vs def2-TZVPP); B3LYP/CPCM-computed vertical and adiabatic ionization energies and inner sphere reorganization energies for PMBA and its oxidized intermediates; spin density isosurfaces along the deprotonation coordinate in both bulk and interfacial environments; density profiles of NaOAc/H_2_O electrolyte near the electrode surface under restrained PMBA configurations; free energy profiles for PMBA cation radical deprotonation by acetate within the 0.4 M NaOAc double layer computed from the second PMF minimum; DFT computed energy surface for deprotonation of PMBA carbocation to the final aldehyde product (PDF)
Example simulation input files and scripts needed to run simulations described in this work (ZIP)
