# Supplementary material for: ECE vs DISP Mechanisms in Anodic Electrolysis of Benzyl Alcohols: Computational Prediction of Microscopic Rate Constants
Source: J Phys Chem C Nanomater Interfaces. 2025 Aug 28;129(36):16076–96. doi: 10.1021/acs.jpcc.5c04347 (PMC12434723; doi:10.1021/acs.jpcc.5c04347)
Supplement: Supplementary file 1 [file jp5c04347_si_001.pdf]

Supplemental Information for "ECE vs DISP Mechanisms in Anodic Electrolysis of Benzyl Alcohols: Computational Prediction of Microscopic Rate Constants"

John H. Hymel and Jesse G. McDaniel*

School of Chemistry and Biochemistry, Georgia Institute of Technology, Atlanta, Georgia 30332-0400, United States

E-mail: mcdaniel@gatech.edu

Contents

S1 System Construction and Force Field Details **S2**

S2 Estimation of Electrode Surface Charge from Applied Potential **S4**

S3 Desorption Rate Constant Calculations **S6**

S4 QM/MM Simulation Protocols **S7**

S5 Lennard Jones Tuning and RDF Benchmarking **S8**

S6 Voronoi Polyhedra Restraint for Excess Proton Localization **S10**

S7 QM/MM Umbrella Sampling Convergence **S11**

S8 QM/MM Free Energy Profiles for PMBA Cation Radical Deprotonation: Basis Set Benchmark, def2-SVP vs def2-TZVPP S14

S9 Vertical and Adiabatic Ionization Energies of PMBA and Its Intermediates S15

S10 Spin Density Analysis S16

S11 NaOAc/H2O Density Profile S20

S12 QM/MM Free Energy Profiles for PMBA Cation Radical Deprotonation by Acetate in the Second Interfacial Minimum S22

S13 Deprotonation of PMBA Carbocation to Aldehyde S23

References S25

S1 System Construction and Force Field Details

Because of finite size effects, the ion concentration in the bulk electrolyte region is depleted with double layer/electrode charging. Thus the appropriate bulk ion concentration of the simulation is reported as the value after the double layer charging is equilibrated for a given set value of electrode surface charge. The computed bulk electrolyte concentrations corresponding to the various electrode surface charge densities studied are given in Figure S1.

Atomic partial charges for both the neutral and cation radical states of PMBA were fit using the approach of Ferenczy and co-workers, based on electrostatic potential fitting to a distributed multipole expansion (DMA).[1–4] Geometry optimizations and single-point calculations were carried out at the PBE0/6-31G* level of theory using Psi4,[5] followed by DMA analysis and least-squares fitting to reproduce the molecular electrostatic potential.

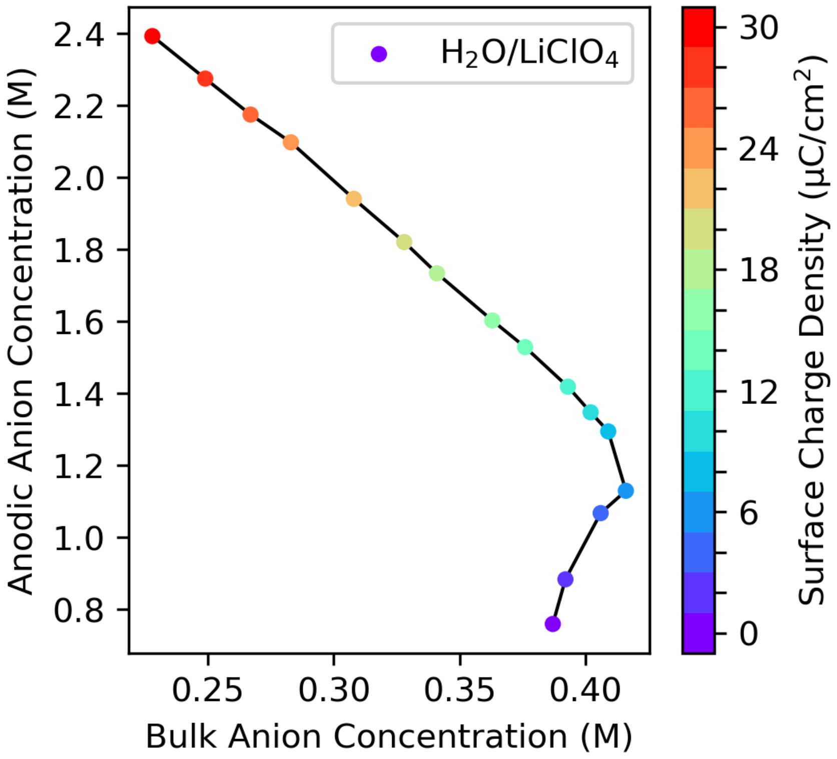

Figure S1: Relationship between bulk perchlorate concentration and anodic double layer perchlorate concentration as a function of electrode surface charge density. The anodic double layer region is defined as the volume within 15 Å of the anode surface. Similarly, the bulk region is defined as the volume 15 Å from either electrode surface.

This charge assignment protocol has been used previously in our group's work on oxidation-state-dependent charge fitting for electrochemical simulations.[6] The resulting atomic charges for both PMBA states are consistent with localized electronic structure changes expected upon oxidation and are provided in Table S1.

Table S1: Atomic partial charges for PMBA in the neutral and cation radical states. Charges were assigned via DMA electrostatic potential fitting at the PBE0/6-31G* level of theory.

**Atom Name**	**Neutral Charge**	**Cation Radical Charge**
C00	-0.05520	-0.08463
O01	-0.30430	-0.21240
C02	0.12800	0.35855
C03	-0.22110	-0.08426
C04	-0.10430	-0.09666
C05	-0.18040	0.12651
C06	0.06030	0.29151
O07	-0.58310	-0.58262
C08	-0.10010	-0.09666
C09	-0.17690	-0.08426
H0A	0.09320	0.14097
H0B	0.09320	0.14097
H0C	0.09320	0.14097
H0D	0.15490	0.11804
H0E	0.15050	0.16125
H0F	0.10290	0.02983
H0G	0.10290	0.02983
H0H	0.41260	0.42378
H0I	0.16560	0.16125
H0J	0.16790	0.11804

S2 Estimation of Electrode Surface Charge from Applied Potential

In order to model the electrostatic environment relevant to electrosynthetic oxidation, we applied fixed surface charge densities to the two graphite electrodes to represent the potential of the working (anodic) and counter (cathodic) electrodes. This constant-charge setup follows

the methodology described in our previous work[6] and relates the electrode charge to the applied potential via the relation:

$$Q = C\Delta V \tag{1}$$

where Q is the surface charge density (in µC/cm^2), C is the differential capacitance of the electrode–electrolyte interface, and ΔV is the applied potential relative to the potential of zero charge (PZC).

Experimentally, the oxidation potential of para-methoxybenzyl alcohol (PMBA) is approximately 1.3 V vs. Ag/$AgNO_3$.[7] Using published reference electrode conversion factors[8,9] and a PZC of approximately –0.2 V vs. Fc/Fc^+ for graphitic carbon in aqueous electrolytes,[10] we estimate the PMBA oxidation potential to be ~1.5 V relative to the PZC. This working potential is illustrated in Figure S2, which relates reference potentials on a common scale.

Capacitance values at electrode interfaces are inherently uncertain and depend on the electrolyte concentration and ion-specific effects. At low concentrations, the diffuse layer (Gouy–Chapman) dominates and yields smaller capacitance, while at higher concentrations (0.1–1 M), the compact (Helmholtz) layer dominates and typical values range from 5–20 µF/cm^2.[11] To account for this uncertainty and assess sensitivity to surface charge, we varied the capacitance systematically across this range.

Applying the relation $Q = C\Delta V$ with $\Delta V = 1.5$ V and C from 0 to 20 µF/cm^2 yields surface charge densities from 0 to 30 µC/cm^2. Sixteen discrete values were selected in 2 µC/cm^2 increments across this range. These values were used in both the classical MD and QM/MM simulation series to probe how the double layer environment modulates adsorption and reactivity.

Each periodic graphene electrode contained 800 carbon atoms. At the highest surface charge density of 30 µC/cm^2, this corresponds to ±0.0492 elementary charges per carbon atom, symmetrically applied to the anode and cathode in the simulation cell.

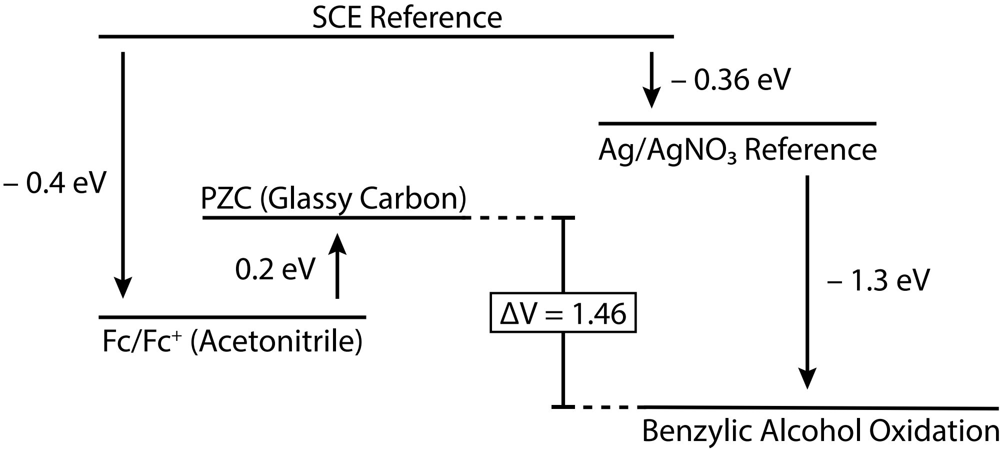

Figure S2: Energy level diagram showing the estimated oxidation potential of PMBA relative to the potential of zero charge (PZC), and its relationship to common reference electrodes. Arrows denote relative energy (opposite in sign to potential).

S3 Desorption Rate Constant Calculations

To predict how long a substrate remains at the electrode surface before diffusing into the bulk electrolyte, we computed desorption rate constants from unbiased classical MD simulations. This approach provides a first-order rate constant, defined as the inverse of the average residence time near the surface.

Our method follows earlier work by Sanchez et al.[12] and Impey et al.,[13] where a collective variable (CV) is tracked over long unbiased trajectories. The time spent in a defined basin (here, the adsorbed state) is measured before crossing a predetermined boundary that marks desorption. The average of these time intervals is then inverted to yield a first-order rate constant (units of s^{-1}).

The desorption process was defined based on PMF minima, which are located at $r_{anode/PMBA} =$ 3.5 Å for neutral PMBA and $r_{anode/PMBA} = 5.0$ Å for oxidized PMBA. The protocol used to define residence periods was:

1. Begin timing once the substrate COM lies within ±0.25 Å of the PMF minimum.

2. Stop timing when the COM reaches 15 Å from the surface.

3. Record the interval as a residence time.
4. Resume timing only after the substrate re-adsorbs (i.e., returns within ±0.25 Å of the minimum).

Simulations were performed for both neutral and cation radical states of PMBA in $LiClO_4$ electrolyte across five surface charge densities: 0, 6, 14, 20, and 28 µC/cm^2. For each condition, ten independent 250 ns unbiased MD simulations were run. At $\sigma = 0$ µC/cm^2, where desorption events were rare, all ten replicas were extended for an additional 250 ns. This resulted in 2.5 µs of sampling per system (5.0 µs for $\sigma = 0$), totaling 30 µs of simulation time across all systems.

S4 QM/MM Simulation Protocols

To evaluate proton transfer free energy barriers under interfacial and bulk conditions, we performed QM/MM molecular dynamics simulations with umbrella sampling using the PyDFT-QMMM package,[14] developed in our group. This package interfaces OpenMM with Psi4 and supports constant-charge electrochemical boundary conditions via classical molecular mechanics modeling of electrodes and solvent.

All simulations were performed at 300 K using Langevin dynamics. The QM region included the PMBA cation radical and one or more proton-accepting solvent molecules, treated at the B3LYP-D3/def2-SVP level of theory.[15,16] For simulations involving water as the proton acceptor, 6–8 QM water molecules were used. For simulations involving acetate, only a single acetate molecule was included in the QM region. The surrounding water, ions, and graphite electrodes were modeled classically using the OPLS-AA force field.[17] Polarization of the QM region by the surrounding environment was treated via electrostatic embedding, with MM partial charges within a 1.4 nm cutoff included in the QM Hamiltonian.

All interfacial simulations were initialized from equilibrated classical MD snapshots at a surface charge density of 14µC/cm^2, representative of anodic working conditions (see Section

S2). For bulk water simulations, a periodic cubic cell containing PMBA and 2000 SPC/E water molecules was used. A representative water or acetate molecule located near the acidic benzylic site of PMBA was selected as the proton acceptor.

Umbrella sampling was performed along a proton transfer coordinate, R_{PT}, defined as the difference between the bond distance from the transferring proton to its donor atom (C_{benz}) and the shortest distance to all QM oxygen atoms (for water) or to the acceptor oxygen (for acetate). This CV is defined as:

$$R_{PT} = d_{C-H^+} - \min(d_{H^+-O})$$

The minimum distance was computed using a smooth, differentiable function to ensure numerical stability during biased MD.

Umbrella windows were initialized using 2 ps steered MD, followed by 16-20 ps of production sampling per window. Two additional restraints were employed for stability: a half-harmonic wall was applied to the non-transferring benzylic hydrogen (at 1.2 Å) to prevent proton exchange, and a Voronoi polyhedron-based CV was used to localize the excess proton when water acted as the base.[18,19] In bulk water simulations, the FIRES restraint[20] was used to prevent MM water molecules from entering the QM solvation shell. Additional sampling details and restraint parameters are provided in the following subsections.

S5 Lennard Jones Tuning and RDF Benchmarking

When transferring a proton from the substrate on to a nearby water molecule, it is important that the transferred proton has force field parameters to a water proton so that its solvation is treated correctly with MM waters. Ensuring this requires adjusting its parameters so that it can properly form hydrogen bonds without causing unphysical configurations. The OPLS-AA benzylic sp^3 hydrogen Lennard Jones (LJ) parameters were intended for a carbon-bound proton that does not engage in hydrogen bonding. Simply switching these parameters

to those of SPC/E water protons introduced a new problem: the oxygen atoms of the classical MM waters, lacking appropriate repulsive interactions, approached the now "water-like" protons too closely. In hydrogen bonding interactions, the LJ parameters of proton donors/acceptors are carefully tuned to prevent nuclei from overlapping. Without a similar adjustment in our system, the proton and oxygen nuclei effectively overlap, causing integrator instability.

To remedy this, we tuned the LJ parameters of the benzylic sp^3 carbon; changing the carbon's σ parameter from 3.5 Å to 5.5 Åand ε from 0.276 kJ/mol to 0.01 kJ/mol. By doing so, we restored a balanced level of repulsion, preventing the water oxygens from coming unphysically close to the newly parameterized protons (now with SPC/E water parameters). After these modifications, we benchmarked the resultant parameter sets against radial distribution functions (RDFs) computed with a single QM PMBA (in the radical cation state) in a cubic box of MM water. We examined three sets of force field parameters: (1) the original OPLS-AA parameters, which behaves correctly when protonated, but lack the proper indistinguishably if a proton was transferred to a QM water; (2) parameters where the benzylic sp^3 hydrogens are replaced with SPC/E water-like protons; and (3) parameters combining SPC/E-like hydrogens with tuned benzylic carbon LJ parameters. These simulations were 10 ps long, except the simulation using parameter set (2), which broke within 500 fs. Three RDFs are compared in Figure S3 using these different sets of force field parameters. Figure S3a shows the RDF between benzylic protons and water oxygens; the curve labeled "SPC/E" (orange) shows the breakdown in the simulations, since the RDF has a peak near zero Å. Comparing the original OPLS-AA RDF (Figure S3a, blue) to that with water parameters and tuned carbon LJ (Figure S3a, green) confirmed that the final parameter set allowed the proton to behave indistinguishably from solvent protons, while maintaining physically realistic proton-oxygen distances and preventing integrator failures. Figures S3b and S3c demonstrate that the modifications to the carbon LJ do not alter the hydrogen bonding interactions of the nearby benzylic alcohol.

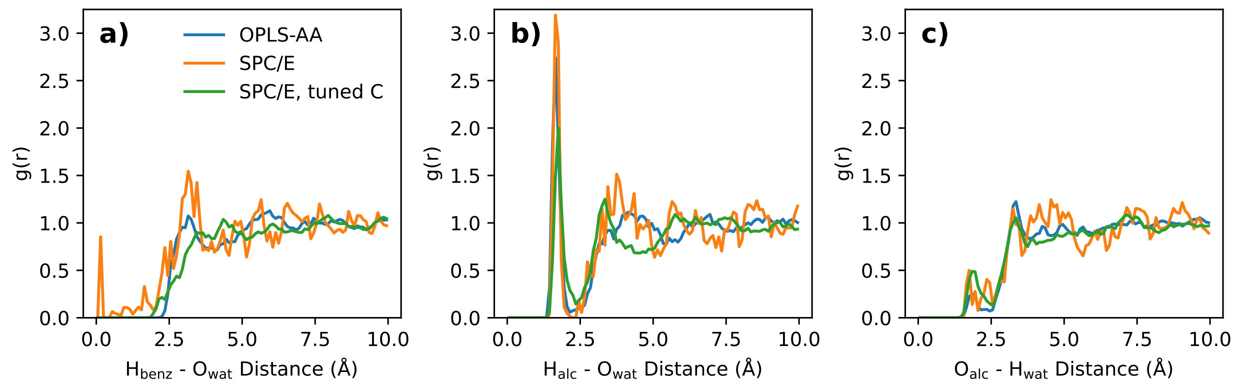

Figure S3: Radial distribution functions (RDFs) comparing interactions between a QM PMBA in the radical cation state and MM water using different force fields. The RDFs are between a) benzylic sp^3 protons (H_{benz}) and water oxygens (O_{wat}), b) benyzlic alcohol proton (H_{alc}) and water oxygens (O_{wat}), and c) benzylic alcohol oxygen (O_{alc}) and water protons (H_{wat}). The "OPLS-AA" label corresponds to the original LJ parameters. The "SPC/E" label corresponds to the forcefield where the benzylic sp^3 hydrogens were switched to use water parameters. The "SPC/E, tuned C" label includes the SPC/E water parameters on hydrogens with the tuned benzylic sp^3 carbon parameters.

S6 Voronoi Polyhedra Restraint for Excess Proton Localization

In simulations of deprotonation by water, the Grotthuss mechanism allows the excess proton to rapidly delocalize across the surrounding solvent network. While the relatively small size of the QM region (6–8 water molecules) limits long-range hopping, this mobility can still interfere with free energy sampling near the transition state. This issue is most pronounced near the transition state, where the transferring proton is weakly associated with both donor and acceptor. Under these conditions, a non-transferring hydrogen atom on the acceptor water may become more labile, initiating undesired proton exchange and driving the system away from the true transition state geometry.

To mitigate this, we employed an auxiliary collective variable (CV) based on the Voronoi polyhedra method developed by the Parrinello group.[18,19] This CV measures the distance between the benzylic sp^3 carbon of PMBA and the excess proton, which is identified through a continuous, differentiable topological criterion. The Voronoi-based CV allows us to track

the location of the excess proton independently of atomic identity, and enables restraining its motion using standard biasing potentials.

We applied a half-harmonic upper wall at 3.5 Å along this CV to prevent the excess proton from drifting too far from the carbon site during umbrella sampling. This restraint was only active when the excess proton attempted to delocalize substantially away from the chosen proton accepting molecule, and had no effect during most of the trajectory. The result is improved stability of the transition state region and more accurate reconstruction of the potential of mean force (PMF). This restraint does not significantly alter the barrier height but helps prevent spuriously low-energy configurations due to unphysical proton delocalization. The application of this restraint was limited to simulations involving water as the proton acceptor. For deprotonation by acetate, the proton remains localized on a single acceptor atom, and no such restraint was necessary.

S7 QM/MM Umbrella Sampling Convergence

To assess convergence of the computed potentials of mean force (PMFs), each umbrella window trajectory was divided into consecutive, non-overlapping 1 ps segments. The WHAM procedure was then applied independently to each set of 1 ps segments, producing a series of PMFs that reflect the evolution of the free energy profile over time. Importantly, these profiles are not cumulative; each is computed using only the data from a distinct time slice of the trajectories. This ensures that each PMF includes an equal and independent amount of sampling from each window.

This time-resolved analysis enables a direct evaluation of convergence. Consistency in the free energy profiles across time intervals indicates that sampling within each window is sufficient and that the PMF has stabilized. In contrast, large variations over time would suggest insufficient equilibration or inadequate overlap between windows. This convergence check provides confidence that the reported activation barriers reflect statistically reliable

sampling across the reaction coordinate.

Figure S4 shows the results of this analysis for four proton transfer reactions studied. The PMFs for deprotonation by water in both bulk and interfacial environments exhibit larger variation with time, particularly in the location and stability of the product well and transition state. This behavior likely reflects the challenge of sampling hydronium solvation configurations. As a result, for reactions involving water as the base, only the second half of each umbrella trajectory was included in WHAM analysis. In contrast, simulations involving acetate exhibited much greater convergence stability, and only the initial 2 ps was discarded from each window.

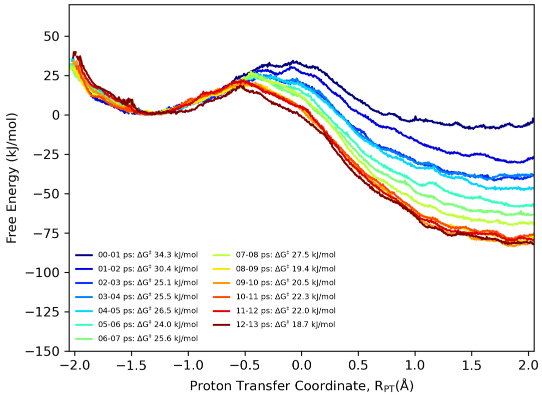

(a) Deprotonation by water in bulk water.

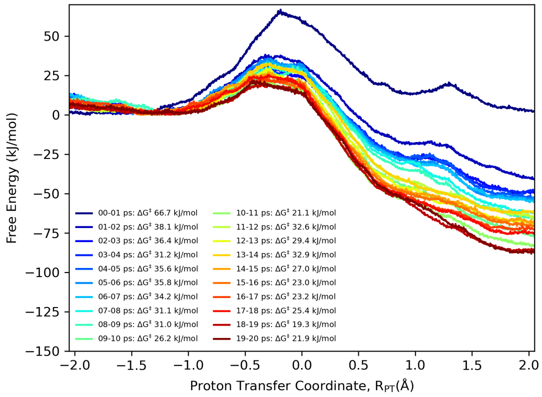

(b) Deprotonation by water at the interface.

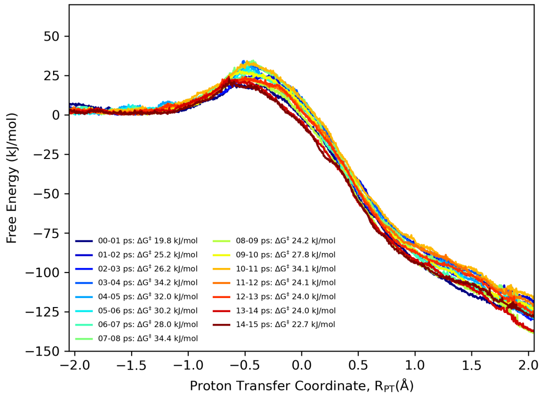

(c) Deprotonation by acetate in bulk water

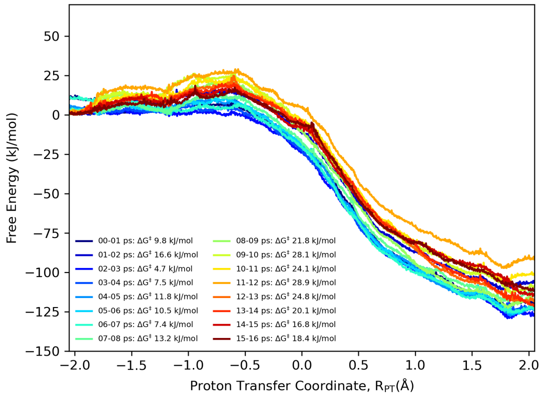

(d) Deprotonation by acetate at the interface (1st Min.)

Figure S4: Time-resolved convergence of the potential of mean force (PMF) for each deprotonation reaction studied. Each PMF is computed using non-overlapping 1 ps segments from the umbrella sampling trajectories. The colors represent successive 1 ps intervals, and the associated activation free energy ($\Delta G^{\ddagger}$) is annotated in each legend. Panels show (a) deprotonation of PMBA by water in bulk water, (b) by water at the electrochemical interface, (c) by acetate in bulk water, and (d) by acetate at the interface (1st Min.).

S8 QM/MM Free Energy Profiles for PMBA Cation Radical Deprotonation: Basis Set Benchmark, def2-SVP vs def2-TZVPP

To assess the sensitivity of computed deprotonation barriers to the choice of basis set, we performed additional DFT-QM/MM umbrella sampling simulations using the larger def2-TZVPP basis set. These simulations were run at the same DFT level of theory (B3LYP-D3) as the primary simulations but used a reduced sampling time of 8 ps per window due to the increased computational cost, compared to 16-20 ps per window for def2-SVP.

A comparison of the resulting free energy profiles for the PMBA cation radical deprotonation reaction is shown in Figure S5. Results shown as computed from the def2-SVP basis set are identical to those given in Figure 9 of the main text for deprotonation to water, in Figure 12 of the main text for deprotonation to acetate. Use of def2-TZVPP systematically increased the computed activation barriers by approximately 10-15 kJ/mol and decreased the overall driving force for deprotonation by a similar amount.

Importantly, the relative ordering of the barriers across all systems was preserved. In particular, deprotonation by acetate at the electrochemical interface continued to exhibit a markedly lower barrier than reactions in bulk or with water as the base. These results support the robustness of our mechanistic conclusions, indicating that the observed effects of the electrical double layer on proton transfer remain qualitatively valid across basis sets.

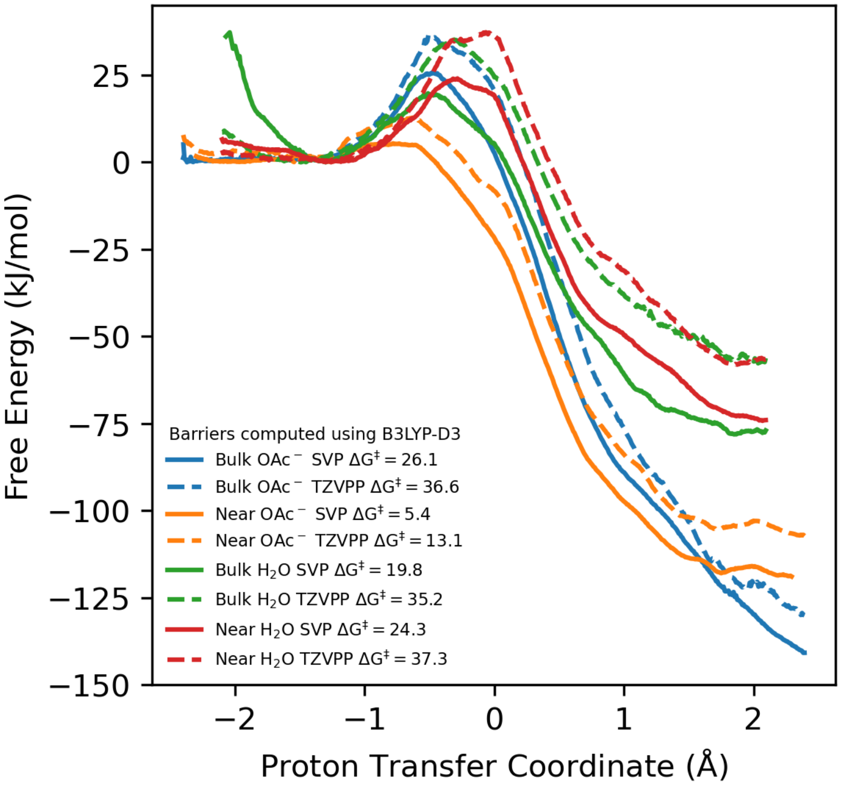

Figure S5: Free energy profiles for PMBA cation radical deprotonation by water and acetate, computed in both bulk water and within $LiClO_4$ or NaOAc double layers at the minimum nearest the electrode surface, using B3LYP-D3/def2-SVP and B3LYP-D3/def2-TZVPP.

S9 Vertical and Adiabatic Ionization Energies of PMBA and Its Intermediates

Vertical and adiabatic ionization energies were computed for para-methoxybenzyl alcohol (PMBA) and its oxidized intermediates using DFT with the B3LYP-D3 functional and two basis sets: def2-SVP and def2-TZVP. All calculations were performed in ORCA 5.0[21] using the conductor-like polarizable continuum model[22] (CPCM) with water as the implicit solvent.

The first ionization energy corresponds to oxidation of the neutral PMBA molecule to the cation radical. The second ionization energy corresponds to oxidation of the benzylic radical (formed after deprotonation) to the carbocation. For each oxidation step, both vertical

ionization energy (VIE) and adiabatic ionization energy (AIE) were computed. Inner sphere reorganization energies (ISR) were calculated using the four-point method.[23]

Table S2: Vertical and adiabatic ionization energies and inner sphere reorganization energies for PMBA and its benzylic radical, computed in implicit solvent using Orca.

**Method**	**VIE (eV)** (0→+•)	**AIE (eV)** (0→+•)	**ISR (kJ/mol)** (0→+•)	**VIE (eV)** (•→+)	**AIE (eV)** (•→+)	**ISR (kJ/mol)** (•→+)
B3LYP-D3/def2-SVP	5.81	5.59	20.47	3.80	3.62	17.19
B3LYP-D3/def2-TZVP	6.04	5.69	28.94	3.89	3.71	17.71

S10 Spin Density Analysis

In the main text, we analyze MBIS atomic charges from simulation snapshots along the deprotonation reaction coordinate for both water- and acetate-mediated pathways. These charges allow us to track the localization of excess positive charge over the course of the reaction. Since all systems are open-shell doublets, a similar analysis can be performed for spin localization by examining the spin density, defined as the difference between αand βelectron densities. Unlike point charges, spin density is a three-dimensional property and cannot be easily assigned to individual atoms. For this reason, we present qualitative visualizations of spin density for representative snapshots corresponding to the reactant, transition state, and product states. These spin densities are shown in Figures S6 and S7 for deprotonation by water and acetate, respectively, in both bulk and interfacial environments. The structures shown are thermalized QM/MM configurations sampled from umbrella sampling simulations, rather than optimized geometries.

Across all systems, the spin density in the reactant state is delocalized across four carbons of the aromatic ring: the three ring carbons adjacent to the methoxy group and the para carbon relative to it. These spin densities correspond well to natural bond orbital (NBO) atomic spin densities computed for similar cation radical para-methyoxybenyzlic species.[24] Following deprotonation, spin density becomes more localized, shifting to the benzylic carbon and three nearby ring carbons, the carbon nearest to the methoxy group and the two carbons

meta to it. This change in distribution suggests a transition from a delocalized radical cation to a more localized aryl radical upon proton loss.

In addition to the ring-localized spin, we observe non-negligible spin density on the benzylic hydrogen atoms in the reactant state. This is consistent with prior findings that spin localization on benzylic hydrogens in aryl radical cations can serve as a predictor of reactivity, particularly for deprotonation or hydrogen abstraction pathways.[25] In their analysis of spin density maps, Hoke and Breton showed that systems exhibiting spin on benzylic hydrogens undergo side-chain substitution or deprotonation, while those lacking such localization favor alternative ring reactivity. Our results support this mechanistic association: the presence of spin density on benzylic hydrogens in the PMBA radical cation correlates with the observed deprotonation pathway and helps rationalize the accessibility of this elementary step across multiple electrochemical environments.

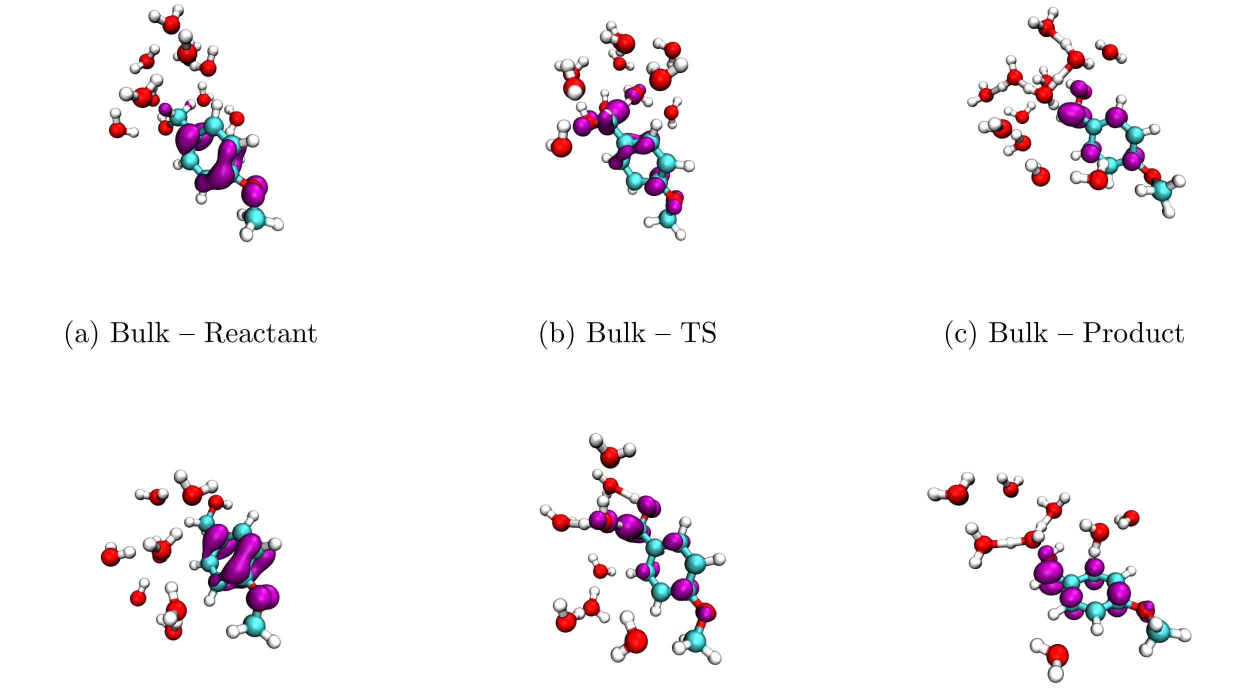

(a) Bulk – Reactant (b) Bulk – TS (c) Bulk – Product

(d) Near Interface – Reactant (e) Near Interface – TS (f) Near Interface – Product

Figure S6: Spin density isosurfaces (isosurface value: 0.005 $e^-/bohr^3$) for doublet-state species in water environments (net +1 charge), comparing bulk and near-interface configurations. Structures correspond to the reactant, transition state, and product. Spin densities were computed using the QM/MM Hamiltonian, and only the QM atoms are shown.

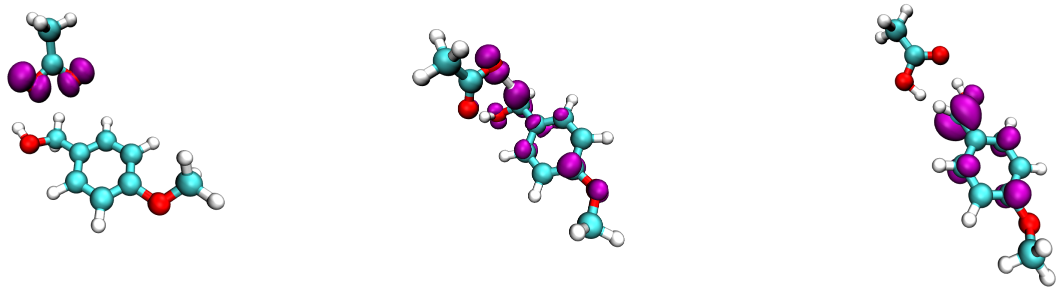

(a) Bulk – Reactant (b) Bulk – TS (c) Bulk – Product

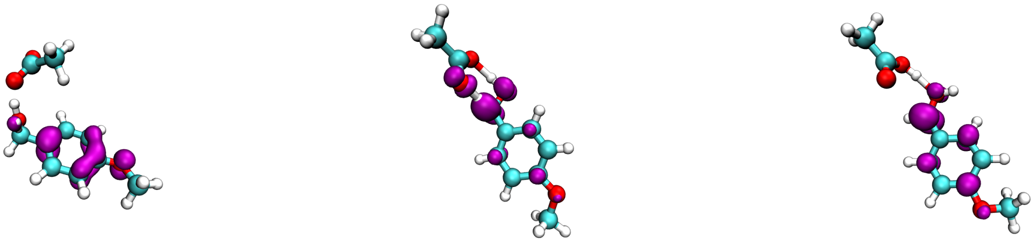

(d) Near Interface – Reactant (e) Near Interface – TS (f) Near Interface – Product

Figure S7: Spin density isosurfaces (isosurface value: 0.005 $e^{-}/bohr^3$) for doublet-state species in acetate environments (net 0 charge), comparing bulk and near-interface configurations. Structures correspond to the reactant, transition state, and product. Spin densities were computed using the QM/MM Hamiltonian, and only the QM atoms are shown.

In the acetate-mediated deprotonation snapshots, the bulk-phase reactant rendering shows unexpected spin density localization on the acetate anion, rather than on the PMBA substrate. This behavior is not observed at the electrode interface, where spin remains centered on PMBA, suggesting that the local environment strongly influences electron distribution. Spin on acetate raises the possibility of a Kolbe-like pathway, in which acetate oxidation precedes decarboxylation. While such mechanisms are typically associated with aliphatic carboxylates under oxidative conditions, the observation suggests that acetate oxidation may be thermodynamically competitive with PMBA oxidation in bulk-like environments. To evaluate this, we computed adiabatic ionization energies in implicit water (Table S3). Across multiple methods, including B3LYP-D3, ωB97X-D3BJ, and MP2, acetate con-

sistently shows a lower or comparable ionization energy than PMBA. This supports the plausibility of transient acetate oxidation in the absence of direct electrode coupling. However, implicit solvent models neglect explicit hydrogen bonding and ion pairing, which can significantly stabilize the acetate anion, particularly near charged interfaces. This likely explains why spin localization reverts to PMBA at the interface. The spin density observed in the QM/MM bulk snapshot, with spin centered on acetate, may therefore reflect a real but transient electron redistribution driven by solvation effects in the absence of electrode stabilization.

Table S3: Adiabatic ionization energies (AIEs, in eV) for PMBA and acetate in implicit water solvent, computed using ORCA. ΔAIE refers to the difference Acetate - PMBA.

**Method**	**Basis Set**	**PMBA AIE (eV)**	**Acetate AIE (eV)**	**ΔAIE (eV)**
B3LYP-D3	def2-SVP	5.59	5.13	-0.46
B3LYP-D3	def2-TZVPP	5.69	5.63	-0.06
ωB97X-D3BJ	def2-SVP	5.88	5.49	-0.39
ωB97X-D3BJ	def2-TZVPP	5.90	5.92	+0.02
MP2	cc-pVTZ	6.49	6.08	-0.41

S11 NaOAc/H2O Density Profile

Figure S8 shows the full density profile of a 0.4 M NaOAc/H_2O electrolyte in the anodic double layer, with the cation radical PMBA restrained 5.5 Å from the electrode surface using an umbrella potential. This configuration corresponds to the "tilted" geometry discussed in the main text, and the simulation was performed at a surface charge density of $\sigma = 14$ µC/cm^2.

The profile includes explicit density-vs-distance curves for water, acetate, and each of PMBA's functional groups, generated from a 20 ns trajectory with data collected every 100 ps. Because this profile is based on a single window containing one PMBA molecule, some noise is expected, and the many overlapping curves can obscure broader trends.

For clarity, a simplified version of this profile was shown in the main text, using shaded

regions to represent the average position of each PMBA functional group. These shaded regions were determined by identifying which group exhibits the highest local density at each distance from the electrode, thereby highlighting the dominant structural motif within the double layer. The full, unprocessed data are provided here for completeness.

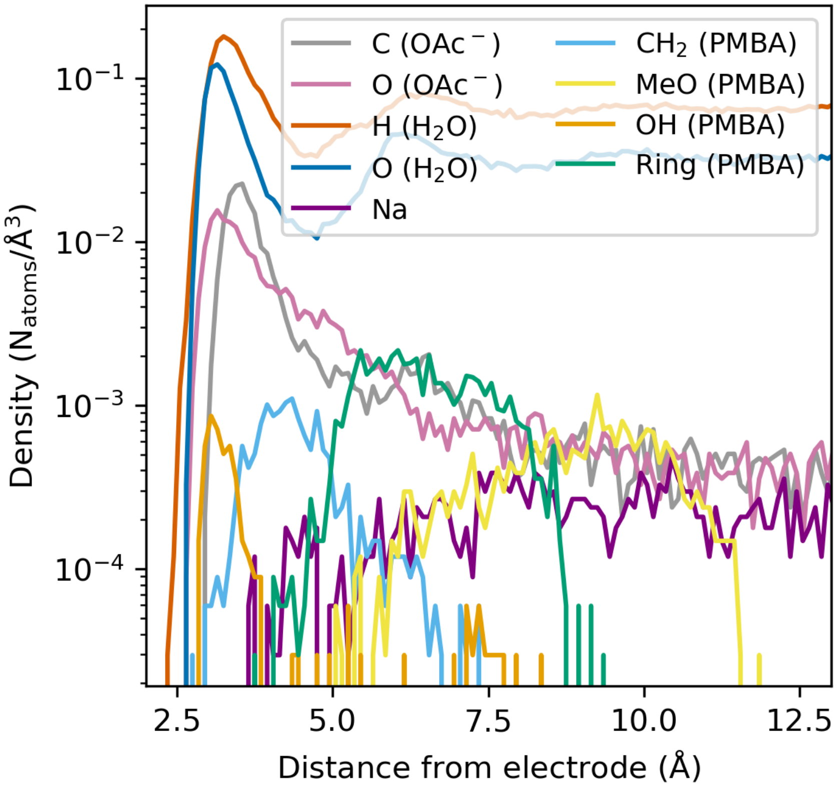

Figure S8: Full density profile of a 0.4 M NaOAc/H_2O electrolyte with σ = 14 μC/cm^2, showing the cation radical PMBA restrained 5.5 Å from the electrode surface by an umbrella potential. This corresponds to the "tilted" configuration discussed in the main text. Density curves are given for water, acetate, and individual PMBA functional groups.

S12 QM/MM Free Energy Profiles for PMBA Cation Radical Deprotonation by Acetate in the Second Interfacial Minimum

A free energy profile was also computed for deprotonation of the PMBA cation radical by acetate within the NaOAc aqueous double layer at the second interfacial solvation minimum, located approximately 7 Å from the electrode surface ("Interface 2nd Min"). This reaction free energy profile was generated using a QM region that included two acetate ions, in contrast to the single-acetate QM region used in the bulk and "Interface 1st Min" simulations. The presence of an additional acetate increases the complexity of the simulation, slows convergence, and introduces additional interactions that complicate direct comparison. This additional "Interface 2nd Min" free energy profile is shown in Figure S9; the "Bulk" and "Interface 1st Min" curves are identical to the free energy profile shown in Figure 12 of the main text. The resulting free energy profile shows a less favorable reaction thermodynamics relative to the bulk and first interfacial minimum PMFs, though this is likely due to the altered simulation setup and sampling challenges.

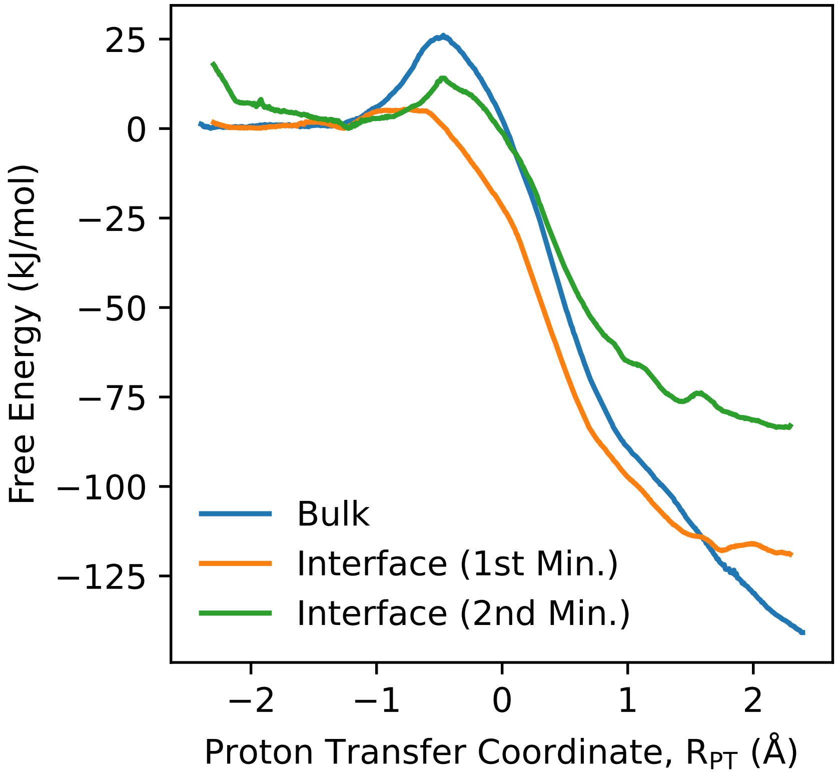

Figure S9: Free energy reaction profile for PMBA cation radical deprotonation by acetate anion in bulk water (blue curve "bulk") and within the anodic double layer at $\sigma = 14$ µC/cm^2 surface charge and 0.4 M NaOAc electrolyte, for the substrate residing within the $\sim$ 5.5 Å "tilted" PMF minimum (orange curve "interface 1st Min."), and substrate residing within the second $\sim$7 Å PMF minimum (green curve "interface 2nd Min."). Free energy profiles are computed with QM/MM at the B3LYP-D3/def2-SVP level of theory.

S13 Deprotonation of PMBA Carbocation to Aldehyde

The final step in the oxidation of para-methoxybenzyl alcohol (PMBA) to the aldehyde product is deprotonation of the PMBA carbocation intermediate. This intermediate is formed via oxidation of the PMBA radical and was assumed to undergo rapid, barrierless deprotonation to yield the aldehyde. To test this assumption, we performed a relaxed potential energy scan along the O–H coordinate corresponding to this deprotonation reaction, varying the O–H

bond length from 1.0 to 1.8 Å in 20 steps.

Calculations were carried out using the ORCA software package (version 6.0.1)[21] at the B3LYP-D3/def2-TZVPP level of theory with implicit solvation (CPCM, water). The simulation included three explicit water molecules: one acting as the proton acceptor and two others stabilizing the resulting hydronium ion through hydrogen bonding.

The resulting potential energy surface, shown in Figure S10, confirms that deprotonation proceeds without an energy barrier. While the carbocation geometry (structure A) does not correspond to a local minimum, it does present a shallow shoulder on the energy profile. This suggests some stabilization of the intermediate before the proton transfer. The lowest-energy structure, labeled B, corresponds to the aldehyde product and a hydronium ion. The barrierless nature of this reaction towards the aldehyde product supports our assumption that this step is fast and not rate-limiting in the overall mechanism.

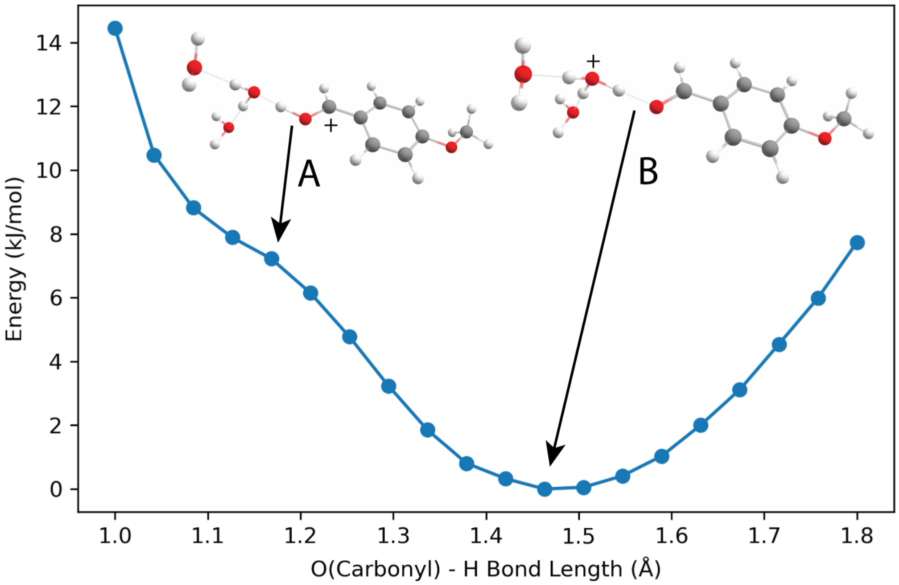

Figure S10: Relaxed potential energy scan for deprotonation of the PMBA carbocation by water to form the aldehyde and a hydronium ion. Structure A represents the carbocation intermediate geometry, while structure B corresponds to the aldehyde product. Calculations were performed at the B3LYP-D3/def2-TZVPP/CPCM(H_2O) level of theory.

References

(1) Chipot, C.; Angyan, J. G.; Ferenczy, G. G.; Scheraga, H. A. Transferable Net Atomic Charges from a Distributed Multipole Analysis for the Description of Electrostatic Properties - A Case-Study of Saturated-Hydrocarbons. *Journal of Physical Chemistry* **1993**, *97*, 6628–6636.

(2) Ferenczy, G. G. Charges Derived from Distributed Multipole Series. *Journal of Computational Chemistry* **1991**, *12*, 913–917.

(3) Stone, A. J.; Alderton, M. Distributed Multipole Analysis - Methods and Applications. *Molecular Physics* **1985**, *56*, 1047–1064.

(4) Stone, A. J. Distributed Multipole Analysis: Stability for Large Basis Sets. *Journal of Chemical Theory and Computation* **2005**, *1*, 1128–1132.

(5) Smith, D. G. A. et al. PSI4 1.4: Open-source software for high-throughput quantum chemistry. *The Journal of Chemical Physics* **2020**, *152*, 184108.

(6) Hymel, J. H.; Khan, S. N.; Pederson, J. P.; McDaniel, J. G. Computational Electrosynthesis Study of Anodic Intramolecular Olefin Coupling: Elucidating the Role of the Electrical Double Layer. *The Journal of Physical Chemistry C* **2023**, *127*, 19489–19508.

(7) Mayeda, E. A.; Miller, L. L.; Wolf, J. F. Electrooxidation of benzylic ethers, esters, alcohols, and phenyl epoxides. *Journal of the American Chemical Society* **1972**, *94*, 6812–6816.

(8) Pavlishchuk, V. V.; Addison, A. W. Conversion constants for redox potentials measured versus different reference electrodes in acetonitrile solutions at 25°C. *Inorganica Chimica Acta* **2000**, *298*, 97–102.

(9) Oyama, M. Formal Potentials in Non-Aqueous Solvents Part I. Organic compounds. *Review of Polarography* **2004**, *50*, 19–42.

(10) Klein, J. M.; Panichi, E.; Gurkan, B. Potential dependent capacitance of [EMIM][TFSI] [N1114][TFSI] and [PYR13][TFSI] ionic liquids on glassy carbon. *Physical Chemistry Chemical Physics* **2019**, *21*, 3712–3720.

(11) Bard, A. J.; Faulkner, L. R. *Electrochemical Methods: Fundamentals and Applications*, 2nd ed.; Wiley, 2001.

(12) Sánchez, H. R. Residence Times from Molecular Dynamics Simulations. *The Journal of Physical Chemistry B* **2022**, *126*, 8804–8812.

(13) Impey, R. W.; Madden, P. A.; McDonald, I. R. Hydration and mobility of ions in solution. *The Journal of Physical Chemistry* **1983**, *87*, 5071–5083.

(14) Pederson, J. P.; McDaniel, J. G. PyDFT-QMMM: A modular, extensible software framework for DFT-based QM/MM molecular dynamics. *The Journal of Chemical Physics* **2024**, *161*, 034103.

(15) Grimme, S.; Antony, J.; Ehrlich, S.; Krieg, H. A consistent and accurate ab initio parametrization of density functional dispersion correction (DFT-D) for the 94 elements H-Pu. *The Journal of Chemical Physics* **2010**, *132*, 154104.

(16) Weigend, F.; Ahlrichs, R. Balanced basis sets of split valence, triple zeta valence and quadruple zeta valence quality for H to Rn: Design and assessment of accuracy. *Phys. Chem. Chem. Phys.* **2005**, *7*, 3297–3305.

(17) Jorgensen, W. L.; Maxwell, D. S.; Tirado-Rives, J. Development and testing of the OPLS all-atom force field on conformational energetics and properties of organic liquids. *Journal of the American Chemical Society* **1996**, *118*, 11225–11236.

(18) Grifoni, E.; Piccini, G. M.; Parrinello, M. Microscopic description of acid–base equilibrium. *Proceedings of the National Academy of Sciences* **2019**, *116*, 4054–4057.

(19) Grifoni, E.; Piccini, G.; Lercher, J. A.; Glezakou, V.-A.; Rousseau, R.; Parrinello, M. Confinement effects and acid strength in zeolites. *Nature Communications* **2021**, *12*, 2630.

(20) Rowley, C. N.; Roux, B. The Solvation Structure of Na+ and K+ in Liquid Water Determined from High Level ab Initio Molecular Dynamics Simulations. *Journal of Chemical Theory and Computation* **2012**, *8*, 3526–3535.

(21) Neese, F. Software Update: The ORCA program system—Version 5.0. *WIREs Computational Molecular Science* **2022**, *12*, e1606.

(22) Barone, V.; Cossi, M. Quantum calculation of molecular energies and energy gradients in solution by a conductor solvent model. *The Journal of Physical Chemistry A* **1998**, *102*, 1995–2001.

(23) Warburton, R. E.; Soudackov, A. V.; Hammes-Schiffer, S. Theoretical Modeling of Electrochemical Proton-Coupled Electron Transfer. *Chemical Reviews* **2022**, *122*, 10599–10650, PMID: 35230812.

(24) Van Lommel, R.; Verschueren, R. H.; De Borggraeve, W. M.; De Vleeschouwer, F.; Stuyver, T. Can the Philicity of Radicals Be Influenced by Oriented External Electric Fields? *Organic Letters* **2022**, *24*, 1–5, PMID: 34652164.

(25) Breton, G. W.; Hoke, K. R. Application of Radical Cation Spin Density Maps toward the Prediction of Photochemical Reactivity between N-Methyl-1,2,4-triazoline-3,5-dione and Substituted Benzenes. *The Journal of Organic Chemistry* **2013**, *78*, 4697–4707, PMID: 23594077.
